# De novo sequencing of tree peony (*Paeonia suffruticosa*) transcriptome to identify critical genes involved in flowering and floral organ development

**DOI:** 10.1186/s12864-019-5857-0

**Published:** 2019-07-11

**Authors:** Shunli Wang, Jie Gao, Jingqi Xue, Yuqian Xue, Dandan Li, Yanren Guan, Xiuxin Zhang

**Affiliations:** 1 Key Laboratory of Biology and Genetic Improvement of Horticultural Crops, Ministry of Agriculture and Rural Affairs, Beijing, People’s Republic of China; 2 Institute of Vegetables and Flowers, Chinese Academy of Agricultural Science, Institute of Peony, Chinese Academy of Agricultural Science, Beijing, 100081 China

**Keywords:** Tree peony, Transcriptome, Flowering induction pathway, Floral model, Re-blooming, MADS-box gene

## Abstract

**Background:**

Tree peony (*Paeonia suffruticosa* Andrews) is a globally famous ornamental flower, with large and colorful flowers and abundant flower types. However, a relatively short and uniform flowering period hinders the applications and production of ornamental tree peony. Unfortunately, the molecular mechanism of regulating flowering time and floral organ development in tree peony has yet to be elucidated. Because of the absence of genomic information, 454-based transcriptome sequence technology for de novo transcriptomics was used to identify the critical flowering genes using re-blooming, non-re-blooming, and wild species of tree peonies.

**Results:**

A total of 29,275 unigenes were obtained from the bud transcriptome, with an N50 of 776 bp. The average length of unigenes was 677.18 bp, and the longest sequence was 5815 bp. Functional annotation showed that 22,823, 17,321, 13,312, 20,041, and 9940 unigenes were annotated by NCBI-NR, Swiss-Prot, COG, GO, and KEGG, respectively. Within the differentially expressed genes (DEGs) 64 flowering-related genes were identified and some important flowering genes were also characterized by bioinformatics methods, reverse transcript polymerase chain reaction (RT-PCR), and rapid-amplification of cDNA ends (RACE). Then, the putative genetic network of flowering induction pathways and a floral organ development model were put forward, according to the comparisons of DEGs in any two samples and expression levels of the important flowering genes in differentiated buds, buds from different developmental stages, and with GA or vernalization treated. In tree peony, five pathways (long day, vernalization, autonomous, age, and gibberellin) regulated flowering, and the floral organ development followed an ABCE model. Moreover, it was also found that the genes *PsAP1*, *PsCOL1*, *PsCRY1*, *PsCRY2*, *PsFT*, *PsLFY*, *PsLHY*, *PsGI*, *PsSOC1*, and *PsVIN3* probably regulated re-blooming of tree peony.

**Conclusion:**

This study provides a comprehensive report on the flowering-related genes in tree peony for the first time and investigated the expression levels of the critical flowering related genes in buds of different cultivars, developmental stages, differentiated primordium, and flower parts. These results could provide valuable insights into the molecular mechanisms of flowering time regulation and floral organ development.

**Electronic supplementary material:**

The online version of this article (10.1186/s12864-019-5857-0) contains supplementary material, which is available to authorized users.

## Background

Tree peony (*Paeonia suffruticosa* Andrews) belongs to section *Moutan* DC of the genus *Paeonia* and family Paeoniaceae and is the first candidate for China’s national flower. Tree peony is valued all over the world due to its large and colorful flowers [1, 2]. There are nine wild species of tree peony, *P. suffruticosa*, *P. cathayana*, *P. jishanensis*, *P. qiui*, *P. ostii*, *P. rockii*, *P. decomposita*, *P. delavayi*, and *P. ludlowii*, and more than 2000 cultivars of *P. suffruticosa* worldwide have been produced using conventional breeding [1–3]. The origin of the most important garden ornamental cultivars in China is a result of homoploid hybridization between *P. ostii*, *P. qiui*, *P. rockii*, *P. jishanensis*, and *P. cathayana* species, while the new varieties with colorful flowers from cultivation of *P. lutea* and *P. suffruticosa* were the result of tree peony breeding breakthroughs since 1997 (Martin, 1997; Zhou et al. 2014). Now, tree peony cultivars can be geographically classified into seven worldwide groups: (1) Chinese Zhongyuan cultivars, (2) Chinese Xibei cultivars, (3) Chinese Xinan cultivars, (4) Chinese Jiangnan cultivars, (5) European cultivars, (6) American cultivars, and (7) Japanese cultivars [1]. Flowering times differ among different cultivars. Generally, the flowering time of Chinese cultivars is earlier than that of Japanese cultivars, and European cultivars and American cultivars are relatively late, having the same flowering time as *P. delavayi* and *P. ludlowii*. The different flowering time and long flowering period are very important for applications and potted production of tree peony. Thus, understanding of the molecular mechanism of flowering time in tree peony could provide a theoretical basis for flowering regulation and breeding.

In *Arabidopsis*, flowering at the right time is ensured by an intricate regulatory network that has evolved in response to a diverse range of environmental and internal signals. More than 80 genes that regulate flowering time have been identified by genetic and physiological analysis of flowering time in *Arabidopsis* [4]. Regulation occurs through well-established flowering genetic pathways, such as photoperiod, vernalization, gibberellins (GA), age, autonomous, and thermosensory pathways [5–8]. *FLOWERING LOCUS T* (*FT*), *SUPPERSSOR OF CONSTANS OF OVEREXPRESSION1* (*SOC1*), and *LEAFY* (*LFY*) are considered integrating factors in these pathways and are located downstream of *FLOWERING LOCUS C* (*FLC*) and *CONSTANS* (*CO*) genes, which regulate flowering time by integrating different flowering signals [8, 9].

Timely flowering determines the commercial value of tree peonies. In the past decade, forcing culture technology and re-blooming in autumn was first investigated to achieve tree peony flowering at the proper time. These studies focused on cultivar selection, physiological change, chilling effect, and hormone analysis [1, 2, 10–12]. The effects of exogenous GA_3_ on flowering quality, endogenous hormones, and hormone- and flowering-associated gene expression in a forcing culture of tree peony were also deciphered [13]. Endo-dormancy-imposed growth arrest is one of the key characteristics preventing tree peony from flowering well. Huang et al. [14] and Gai et al. [15] used a subtractive cDNA library and transcriptome sequencing, respectively, to identify key genes associated with the release of dormant buds in tree peony; genes included *PsII*, *PsMPT*, *GA2*, *GA20ox*, *GA2ox*, *RGA1*, *SPINDLY* (*SPY*), and *AMY2*. *PsFT*, *PsVIN3*, *PsCO*, and *PsGA20ox* were identified to play important roles in the regulation of re-blooming in tree peony by transcriptome sequencing [16]. According to the reported transcriptome results, some functional genes related to flowering, including *SHORT VEGETATIVE PHASE* (*SVP*), *SQUAMOSA PROMOTER BINDING PROTEIN LIKE 9* (*SPL9*), and *SOC1*, have also been cloned [1, 2, 12]. However, the detailed mechanism of the flowering induction pathway is unclear in tree peony, which affects the improvement of the quality of the forcing culture of tree peony.

RNA-seq is a recently developed approach for profiling transcriptomes [17] that has many advantages including being cost-effective, highly sensitive, accurate, and having a large dynamic range. Due to these advantages, RNA-seq is now widely used to analyze gene expression, discover novel transcripts, decipher the molecular mechanism of regulated development and growth, and develop SNP and SSR markers [16–23]. In particular, it has been a powerful tool for analysis of species that lack reference genome information [24].

In this study, we described the utilization of 454-based transcriptome sequencing technology for de novo transcriptomics to identify the critical flowering-related genes using reblooming, non-re-blooming, and wild species of tree peonies. We obtained 29,275 unigenes, including 64 flowering-related genes, and proposed a flowering induction pathway and floral organ development model by analysis of differentially expressed genes (DEGs) between any two samples. Then, the critical flowering-related genes were also selected to do expression analysis in different tree peony cultivars, and buds at different developmental stages or under different treatments; the results validated the postulated flowering induction pathway and floral organ development model. At the same time, ten candidate re-blooming genes were also identified. Our results provide valuable insights into the molecular mechanisms of flowering time regulation and floral organ development of tree peony.

## Results

### 454 GS-FLX sequencing and a de novo assembled tree peony transcriptome

Using 454 sequencing, 31,505 contigs with 20,667,433 total residues were obtained. These contigs were further assembled into 29,275 unigenes, with 19,824 total residues of 498 bp and an N50 of 776 bp. The average length of unigenes was 677.18 bp, and the longest sequence was 5815 bp. The sequence length distribution of the unigenes is shown in Additional file 1: Figure S1. Nearly half of the unigenes (49.03%) ranged from 400 to 600 bp. The GC percentage was 42.73%. All reads were deposited in NCBI and can be accessed in the Short Read Archive (SRA) under accession number SRX863944.

### Functional annotation of tree peony transcriptome

We performed BLASTx (version 2.2.21) analysis against several protein databases: NCBI non-redundant (NR) protein, Swiss-Prot, Clusters of Orthologous Groups (COG), Gene Ontology (GO), and Kyoto Encyclopedia of Genes and Genomes (KEGG) using a cut off E-value of e-5 to annotate tree peony transcriptome. A total of 22,823 unigenes (77.97%) were annotated in the NCBI-NR database based on sequence homology; 17,321 (59.17%) were annotated in Swiss-Prot; 13,312 (45.47%) were annotated in COG; 20,041 (68.46%) were annotated in GO; and 9940 (43.55%) were annotated in KEGG. In addition, 8070 (27.57%) of the unigenes were annotated in the Pfam database. A total of 1939 unigenes were annotated in all databases, while 23,332 unigenes (79.7%) were annotated in at least one database. It was found that the functional annotation of the 5815 bp unigene was 26S ribosomal RNA gene. The detailed results for annotation of the tree peony unigenes are summarized in Table 1.

**Table 1:** The annotations of tree peony bud unigenes against the public databases

Database	NR	Swiss-Prot	COG	GO	KEGG	Pfam	All
Number annotated	22,823	17,321	13,312	20,041	9940	8070	29,275
Percentage (%)	77.97%	59.17%	45.47%	68.46%	34.06%	27.57%	100

**Fig. 1:**
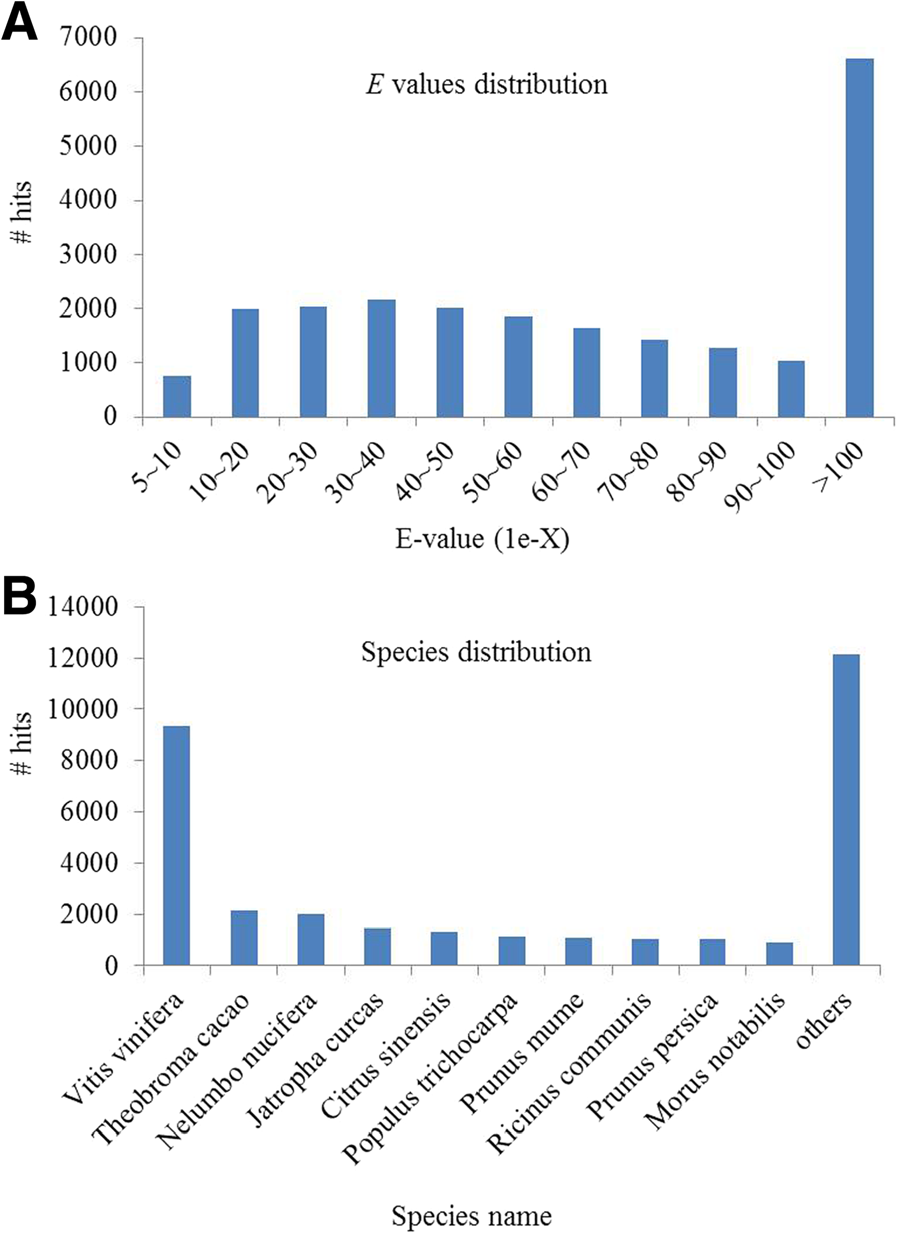
Statistics of homology search of unigenes against NR database. **a** E-value distribution of the top BLASTx hits with a cut-off e-value of 1e-05. **b** Species distribution of the ten top BLASTx hits

Among the unigenes, 10,507 (33.35%) unique sequences shared significant similarity with their matched sequences with an *E* value ranging from *1E*-60 to *1E*-10. Only 30 (0.13%) unique sequences shared weak similarity with the matched sequences (*E* value between 1*E*-180 and 1*E*-190) (Fig. 1a). Further analysis showed that the annotated sequences were matched to sequences of 520 species. Among them, the highest matched species was *Vitis vinifera* and the matched unigenes were 9362 (27.84%). The other top nine species were as follows: *Theobroma cacao* (6.42%), *Nelumbo nucifera* (5.98%), *Jatropha curcas* (4.28%), *Citrus* × *sinensis* (5.80%), *Populus trichocarpa* (3.38%), *Prunus mume* (3.27%), *Ricinus communis* (3.13%), *Prunus persica* (3.05%), and *Morus notabilis* (2.65%) (Fig. 1b).

**Fig. 2:**
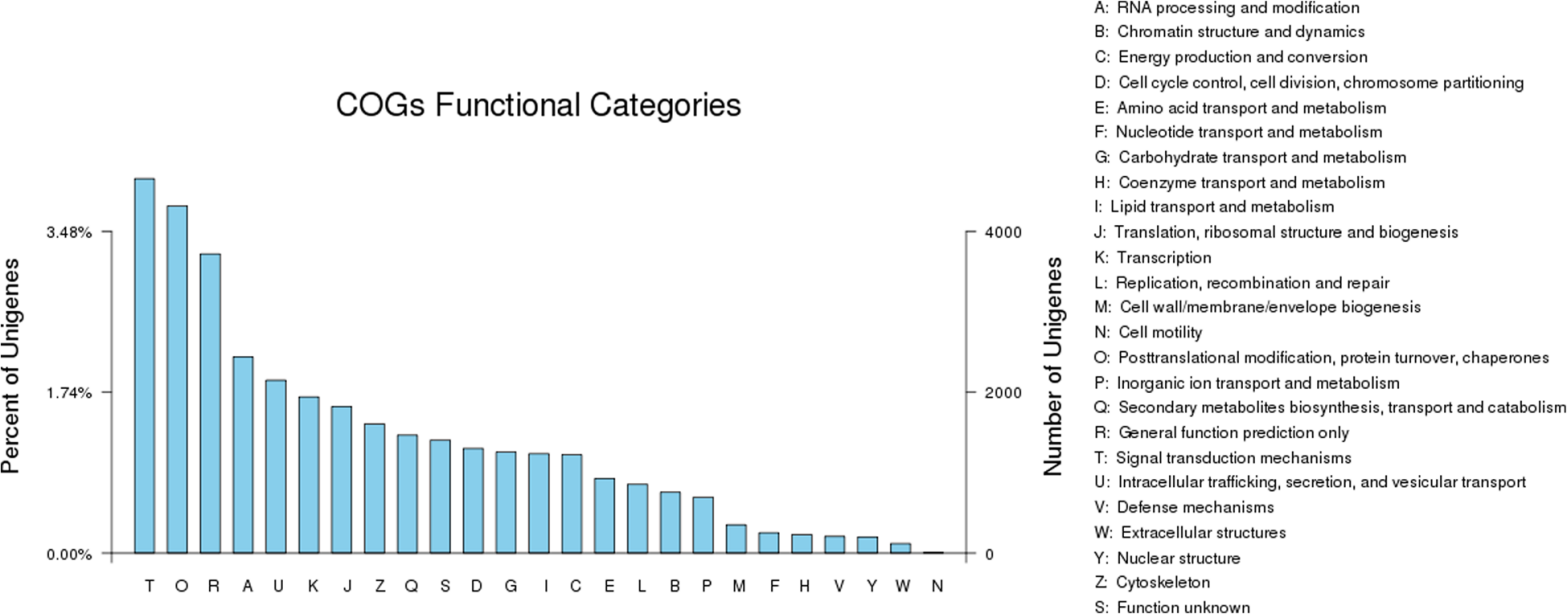
COG functional classification of the tree peony bud transcriptome

To construct a shared protein domain with specific functions, 13,321 unigenes were grouped into 25 functional classifications based on the COG databases (Fig. 2). ‘Signal transduction’ was dominant (13.27%), and the other top three functional groups were ‘Posttranslational modification’ (12.30%), ‘General function prediction only’ (10.61%), and ‘RNA processing and modification’ (6.95%), respectively. ‘Intracellular trafficking, secretion, and vesicular transport’, ‘Transcription’, and ‘Translation, ribosomal structure and biogenesis’ shared 6.12, 5.54, and 5.19% genes among the categories, respectively. The lowest matched term was ‘Cell motility’ and only had 0.017% corresponding genes.

**Fig. 3:**
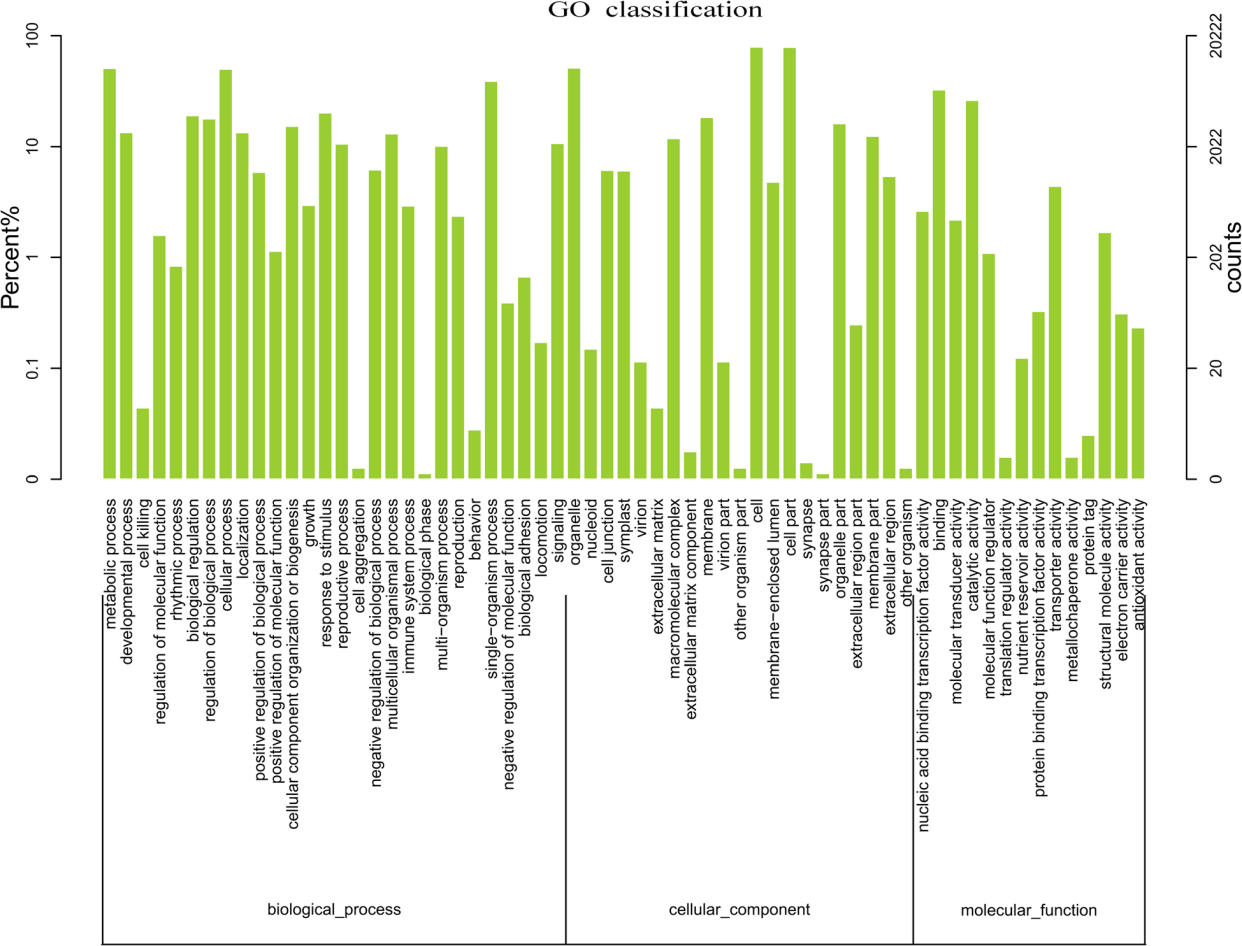
GO classification of the tree peony bud transcriptome

The GO system alignment showed that these unigenes were classified into 63 main functional groups, belonging to biological process, cellular component, and molecular function, respectively (Fig. 3). In biological process, the vast majority was related to metabolic process, cellular process, and single-organism process. In cellular component, genes for cell, cell part, and organelle were the top three. Among the molecular function category, the majority of the GO terms were grouped into binding, catalytic activity, and transporter activity. The detailed information on the annotations was in Fig. 3.

**Fig. 4:**
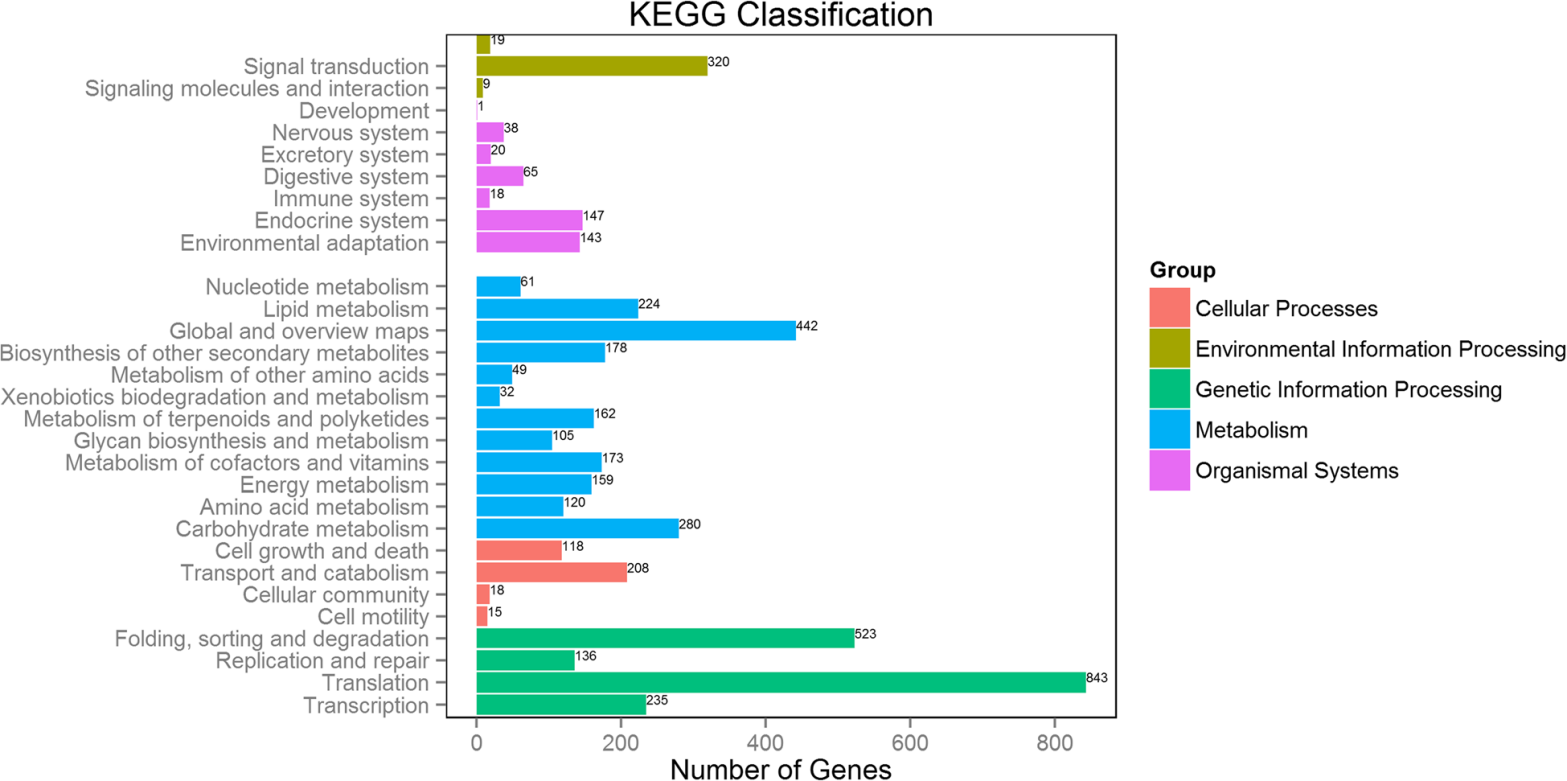
KEGG classification of the tree peony bud transcriptome

Based on KEGG pathway mapping, we annotated and mapped 237 pathways for 9940 unigenes. A summary of the findings is presented in Fig. 4 and Additional file 2: Table S1. The largest number of sequences were those associated with metabolic pathways (1123, 11.30%), followed by sequences that were involved in the biosynthesis of secondary metabolites (557, 5.045%) and biosynthesis of antibiotics (276, 2.78%). In particular, the plant circadian rhythm pathway was obtained using the KEGG database, and 26 genes were identified using the bud transcriptome (Additional file 3: Figure S2). It was suggested that the circadian rhythm was probably important for tree peony flowering.

### Differentially expressed genes (DEGs) identification and analysis through quantitative RNA-seq

Investigating the gene expression level differences between different cultivars or the same cultivar in different developmental stages required identification of DEGs between any two samples. Expression levels of unigenes were determined by aligning the RNA-seq reads from each library to the assembly. A *P*-value < 0.01, FDR ≤ 0.001, and log2 (fold change) ≥ 2 or ≤ − 2 were used as thresholds to identify significant differences between two samples. Comparisons of gene expression in eight groups showed that 1297, 1348, 1484, 1395, 1636, 1058, 1383 and 1489 genes were differentially expressed in ‘Huchuan Han’ (HCH) vs ‘High Noon’ (HN), HCH vs ‘Ziluo Lan’ (ZLL), HCH vs *Paeonia delavayi* (PD), HCH vs ‘Luoyang Hong’ (LYH), HN vs PD, ZLL D (bud at stage D) vs ZLL, ZLL E (bud at stage E) vs ZLL, and ZLL E vs ZLL D, respectively. The detailed information of DEGs in eight groups is shown in Additional file 4: Figure S3, and the unigenes involved in different pathways are in Additional file 2: Table S1. The number of DEGs was largest in HN vs PD and smallest in ZLL D vs ZLL. The possible reason was that HN is a tree peony hybrid (*P. lutea* x *P. suffruticosa*). The most up-regulated genes were in HCH vs ZLL, while there were the fewest up-regulated genes in HCH vs PD. The most down-regulated genes were in HCH vs PD, while the fewest down-regulated genes were in HCH vs ZLL (Additional file 4: Figure S3).

Further analysis of the up-regulated and down-regulated genes in data from eight groups showed that flowering time genes, metabolism genes, and hormone synthesis and signal transduction genes had differential expression in different cultivars or developmental stages. Considering the flowering time character of four cultivars and one wild species, the flowering related genes were investigated further (Additional file 5: Table S2). In HCH vs HN, *SVP*, *CONSTANS-LIKE 1* (*COL1*), *VERNALIZATION INSENSITIVE 3* (*VIN3*), and *AGAMOUS-LIKE 15* (*AGL15*) were down-regulated, while *SPL5*, *GID2, ULTRAPETALA 1*, and *COL4* were up-regulated. At the same time, *FRIGIDA* (*FRI*), *blue-light photoreceptor PHR2*, and *gibberellin receptor GID1a* genes appeared in both the down-regulated and up-regulated groups. In HCH vs PD, *COL1*, *APETALA2* (*AP2*), *PHR2*, *COL16*, and *SVP* were down-regulated, while *FCA* and *GID1* were up-regulated. *FRI* and *GID1a* genes appeared in both down-regulated and up-regulated groups. In HCH vs ZLL, the *PHR2*, *SPL12*, and *GIGANTEA* (*GI*) genes were down-regulated, while the *Phytochrome E* (*PhyE*), *FRI*, and *AGL80* had up-regulated expression. In LYH vs HCH, the *SPL12*, *SPL14*, and *Casein Kinase II* (*CKII*) genes were down-regulated, while *AGL15*, *VIN3*, *EARLY Flowering 3*, *SPL16*, and *COL14* genes were up-regulated. *FRI* appeared in both down-regulated and up-regulated groups. In ZLL vs ZLL D, the *AGL8* and *AGL9* genes were down-regulated, while the *FRI*, *CKII*, and *AGL80* genes were up-regulated. In ZLL vs ZLL E, *GI*, *SPL14*, *LHY*, and *AGL9* were down-regulated, while the *SPL14*, *FRI*, and *CKII* genes were up-regulated. In ZLL D vs ZLL E, the *AGL8* and *SPL9* genes were down-regulated, while the *FRI* and *COL4* genes were up-regulated. In HN vs PD, the *GID1a*, *PhyE*, *AGL15*, and *SPL12* genes were down-regulated, while *FRI* and *COL11* were up-regulated. The *COL4* gene was in both down-regulated and up-regulated groups. *COL1*, *VIN3*, and *PsGI* were the candidate re-blooming genes.

### Identification of putative genes involved in flowering time regulation

Unlike in other model plants, the genetic network of flowering for tree peony is unclear. To identify the transcripts putatively involved in flowering time, flower meristem identity and flower organ identity of tree peony, previously reported flowering related genes in other model plant species, such as *Arabidopsis thaliana*, were used to search the transcripts database. In total, 64 flowering genes were identified in this work (Table 2). In addition, 13 important flowering genes with short sequences (length less than 200 bp) or those not identified by transcriptome sequencing were also isolated using bioinformatics methods, reverse transcript polymerase chain reaction (RT-PCR), and rapid-amplification of cDNA ends (RACE) (Table 2). These genes included flower organ identity genes (class A: *AP1* and *AP2*, class B: *AP3* and *PI*, class C: *AG*, and class E: *AGL9*, *SEP1*, *SEP3*, and *SEP4*); floral integrator pathway genes related to *FT*, *LFY*, and *SOC1*; floral meristem identity genes *CAL* and *AP1*; vernalization pathway genes related to *HOS1-like*, *VIN3*, *VRN1*, and *VRN2*; age pathway gene *SPL9*; GA pathway genes *GAI*, *GID1*, and *SVP*; autonomous pathway gene *FLD*; multiple genes responding to the photoperiod pathway, including *CO*, *COL4*, *COL6*, *COL9*, *CRY1*, *CRY2*, *ELF3*, *ELF4*, *FKF1*, *LHY-like*, *PHYA*, *PHYB*, *PHYC*, *PHYE*, *WNK1*, and *ZTL*; and floral repressor and promoter genes *FRI*, *TFL*, *AG*, and *MAF-like*.

**Table 2:** The identified candidate genes involved in flowering and floral organ development of tree peony

No.	Tree peony gene	Unigene ID	Length	Pathway/Function	*Arabidopsis* gene	*Arabidopsis* GenBank No.	*Arabidopsis* sequence length
1	*PsAG*	Cloned in our lab	1072	Class C floral homeotic gene	*AG* (*AGAMOUS*)	NM_001203837.1	717
2	*PsAGL8*	unigene 5440	812	others	*AGL8* (*AGAMOUS-Like 8*)	NM_125484.4	729
3	*PsAGL9*	unigene 932	1372	Class E floral homeotic gene	*AGL9*	AF015552.1	756
4	*PsAGL14*	unigene 6460	456	others	*AGL14*	NM_001340739.1	666
5	*PsAGL15*	unigene 706	1096	Photoperiod	*AGL15*	NM_121382.3	807
6	*PsAGL17*	unigene 16,415	516	others	*AGL17*	NM_127828.3	684
7	*PsAGL18*	unigene 17,893	486	Photoperiod	*AGL17*	AF312663.1	721
8	*PsAP1*	Cloned in our lab	729	Class A floral homeotic gene	*AP1* (*APETALA1*)	Z16421.1	768
9	*PsAP2*	unigene 16,681	1335	Class A floral homeotic gene	*AP2* (*APETALA2*)	NM_001204009.1	1299
10	*PsAP3*	unigene 17,545	668	Class B floral homeotic gene	*AP3* (*APETALA3*)	AY142590.1	699
11	*PsCAL*	Cloned in our lab	928	Floral meristem identity gene	*CAL* (*CAULIFLOWER*)	NM_102395.2	768
12	*PsCKII*	unigene14187	402	Photoperiod/Circadian clock	*CK2* (*CASEIN KINASE 2*)	BT000888.1	1212
13	*PsCO*	Cloned in our lab	1973	Photoperiod	*CO* (*CONSTANS*)	NM_121589.1	1122
14	*PsCOL1*	unigene 5885	1453	Photoperiod	*COL1*	Y10555.1	1068
15	*PSCOL4*	Cloned in our lab	1125	Photoperiod	*COL4*	NM_122402.3	1221
16	*PsCOL6*	unigene 9556	456	others	*COL6*	AY081541.1	1221
17	*PsCOL9*	unigene 27,384	542	Photoperiod	*COL9*	NM_111644.5	1119
18	*PsCOL10*	unigene 23,649	405	others	*COL10*	NM_124200.3	1122
19	*PsCOL13*	Cloned in our lab	236	others	*COL13*	NM_130356.5	999
20	*PsCOL14*	unigene 5655	1213	others	*COL14*	NM_201860.2	1206
21	*PsCOL16*	unigene 20,771	827	others	*COL16*	NM_102355	1254
22	*PsCRY1*	unigene 5046	2118	Photoperiod/Light perception	*CRY1* (*CRYPTOCHROME 1*)	NM_116961	2046
23	*PsCRY 2*	unigene 13,974	1211	Photoperiod/Light perception	*CRY2*	U43397.1	1839
24	*PsELF3*	unigene 25,740	1171	Photoperiod/Circadian clock	*ELF3* (*EARLY FLOWERING 3*)	NM_128153.2	2088
25	*PsELF 4*	unigene 15,888	615	Photoperiod/Circadian clock	*ELF4*	NM_129566.2	336
26	*Ps ESD4*	unigene 17,002	1053	Autonomous	*ESD4*	AJ582719.1	1470
27	*PsFCA*	unigene 18,962	1159	Autonomous	*FCA*	NM_179211.2	1518
28	*PsFKF1*	unigene 14,676	1059	Photoperiod	*FKF1* (*FLAVIN-BINDING, KELCH REPEAT*, *F-BOX 1*)	NM_105475.3	1860
29	*PsFLC*	unigene 16,860	522	Floral repressor	*FLC* (*FLOWERING LOCUS C*)	AF537203.1	591
30	*PsFLD*	unigene 21,343	221	Autonomous	*FLD* (*FLOWERING LOCUS D*)	AY849996.1	2370
31	*PsFPA*	unigene 10,623	605	Autonomous	*FPA*	NM_129902.2	2577
32	*PsFRI*	unigene 3714	2955	Floral repressor	*FRI* (*FRIGIDA*)	DQ167445.1	1836
33	*PsFT*	unigene 6901	704	Integrator/Floral promoter	*FT* (*FLOWERING LOCUS T*)	AB027504.1	528
34	*FY-like*	Cloned in our lab	2867	Autonomous	*FY*	NM_001203373.1	1962
35	*PsGAI*	unigene 1563	2067	GA	*GAI* (G*IBBERELLIC ACID INSENSITIVE*)	NM_101361.2	1602
36	*PsGI*	unigene 20	4423	Photoperiod	*GI* (*GIGANTEA*)	AF105064.1	3522
37	*HOS1-like*	Cloned in our lab	3062	Vernalization/Cold signalling	*HOS1*(*HIGH EXPRESSION OF OSMOTICALLY RESPONSIVE GENES 1*)	NM_129540.5	2784
38	*PsLD*	unigene 11,537	543	Autonomous	*LD (LUMINIDEPENDENS)*	GQ177537	5936
39	*PsLFY*	unigene 19,888	431	Integrator	*LFY* (*LEAFY*)	NM_125579.1	1263
40	*PsLHY-like*	unigene 15,485	2083	Photoperiod/Circadian clock	*LHY* (*LATE ELONGATED HYPOCOTYL*)	NM_179237.1	1938
41	*PsMAF-like*	unigene 25,853	548	Floral repressor	*MAF-like* (*MADS AFFECTING FLOWERING LIKE*)	NM_126053	624
42	*PsPHYA*	unigene 26,017	1232	Photoperiod/Light perception	*PHY-A* (*PHYTOCHROME A*)	NM_100828	3369
43	*PsPHYB*	unigene 21,416	730	Photoperiod/Light perception	*PHY-B*	EU352781	3507
44	*PsPHYC*	unigene 26,767	787	Photoperiod/Light perception	*PHY-C*	JF318768	3336
45	*PsPHYE*	unigene 17,658	1021	Photoperiod/Light perception	*PHY-E*	NM_117923.7	3339
46	*PsPI*	unigene 1017	862	Class B floral homeotic gene	*PI* (*PISTILLATA*)	JQ180310.1	627
47	*PsPIE1*	unigene 17,987	1944	Floral repressor	*PIE1* (*PHOTOPERIOD-INDEPENDENT EARLY FLOWERING 1*)	AY279398.1	6168
48	*PsPIF3*	Cloned in our lab	3632	Light signaling	*PIF3* (*PHYTOCHROME INTERACTING FACTOR 3*)	AF100166.1	1575
49	*PsPIF1*	unigene 24,639	606	others	*PIF1* (*PHYTOCHROME INTERACTING FACTOR 1*)	NM_001202630.2	1437
50	*PsRGA-like*	unigene 19,948	1331	GA	*RGL* (*REPRESSOR OF GA Like*)	AY048749.1	1536
51	*PsSEP1*	unigene 11,191	512	Class E floral homeotic gene	*SEP1* (*SEPALLATA 1*)	NM_001125758.2	789
52	*PsSEP3*	Cloned in our lab	413	Class E floral homeotic gene	*SEP3*	NM_102272.4	756
53	*PsSEP4*	Cloned in our lab	588	Class E floral homeotic gene	*SEP4*	NM_201682.3	564
53	*PsSOC1*	unigene 13,388	909	Integrator	*SOC1* (*SUPPRESSOR OF OVEREXPRESSION OF CONSTANS1*)	NM_130128.3	645
54	*PsSPL9*	unigene 2914	1552	Age	*SPL9* (*Squamosa promoter binding protein-like 9*)	AJ011639.1	1122
55	*PsSVP*	unigene 327	1239	Floral repressor	*SVP* (*SHORT VEGETATIVE PHASE*)	NM_001161056.1	708
56	*PsTEM1*	unigene 17,174	614	Photoperiod	*TEMPRANILLO (TEM1)*	NM_102367.3	1086
57	*PsTFL1*	Cloned in our lab	582	Others	*TFL1* (*TERMINAL FLOWER 1*)	AF466816.1	534
58	*PsTFL2*	unigene 17,811	1213	Floral repressor	*TFL2* (*TERMINAL FLOWER 2*)	AB073490.1	1338
59	*PsTOC1*	unigene 27,722	1108	Photoperiod/Circadian clock	*TOC1* (*TIMING OF CAB 1*)	AF272039.1	1857
60	*PsTOE1*	unigene 16,681	1335	Putative floral repressor	*TOE1* (*TARGET OF EAT 1*)	NM_128415.4	1350
61	*PsTOE2*	unigene 17,814	514	Putative floral repressor	*TOE2* (*TARGET OF EAT 2*)	NM_001203647.1	1524
62	*PsULT1*	unigene10486	553	Flower development	*ULT1* (*ULTRAPETALA1*)	NM_118959.5	714
63	*PsVIN3*	Cloned in our lab	1689	Vernalization	*VIN3* (*VERNALIZATION INSENSITIVE 3*)	KC505474.1	1863
64	*PsVRN1*	unigene 30,203	428	Vernalization	*VRN1* (*VERNALISATION 1*)	AF289052.1	1026
65	*PsVRN2*	Cloned in our lab	2615	Vernalization	*VRN2* (*VERNALISATION 2*)	AF284500.1	1338
66	*WNK1*	unigene 326	2411	Photoperiod/Circadian clock	*WNK1* (*WITH NO LYSINE (K) KINASE 1*)	NM_001035560.1	2034
67	*PsZTL*	unigene 14,676	1059	Photoperiod/Circadian clock	*ZTL* (*ZEITLUPE*)	AF254413.1	1830

### Relative expression analysis of DEGs related to flowering in the buds of four tree peony cultivars and one wild species

**Fig. 5:**
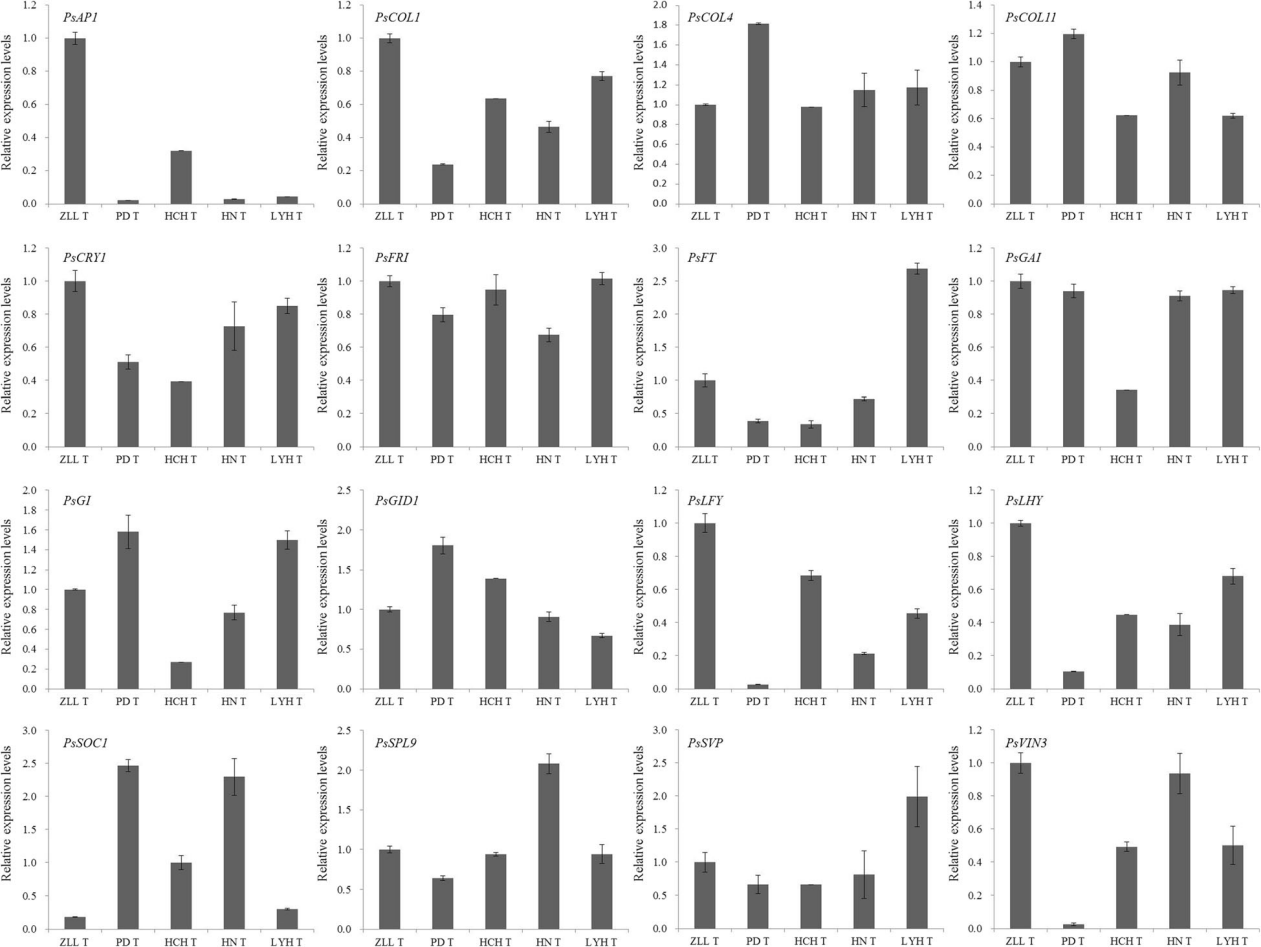
The expression level validation of 12 DEGs in the buds of four cultivars and one wild species by qRT-PCR. ZLL T, PD T, HCH T, HN T, and LHY T represent the five samples used for transcriptome sequencing

To validate the results obtained from the differential gene expression and to determine the potential roles of the flowering genes referred above, we confirmed their expression in the buds of four cultivars and one wild species by qRT-PCR. Expression patterns of most of the DEGs were consistent with those obtained by RNA-seq, confirming the accuracy of the RNA-seq results reported in this study (Fig. 5, Additional file 5: Table S2). Those genes, including *AP1*, *COL1*, *CRY1*, *GAI*, *LFY*, *LYH*, and *VIN3* had high expression in ‘Ziluo Lan’, which easily re-blooms in autumn, together with leaf removal and GA_3_ application treatments. Genes including *FT* and *SVP* had high expression in ‘Luoyang Hong’, which does not easily flower in autumn. *SOC1* and *SPL9* had high expression in ‘High Noon’ which flowers in autumn under natural conditions. Combining the flowering characters of five tree peony cultivars, *AP1*, *COL1*, *CRY1*, *FT*, *GI*, *LFY*, *LYH*, *SOC1*, *SPL9*, *SVP*, and *VIN3* were shown to be associated with tree peony autumn flowering or re-blooming. It was deduced that tree peony flowering was regulated by GA, age, long day, and vernalization pathways.

**Fig. 6:**
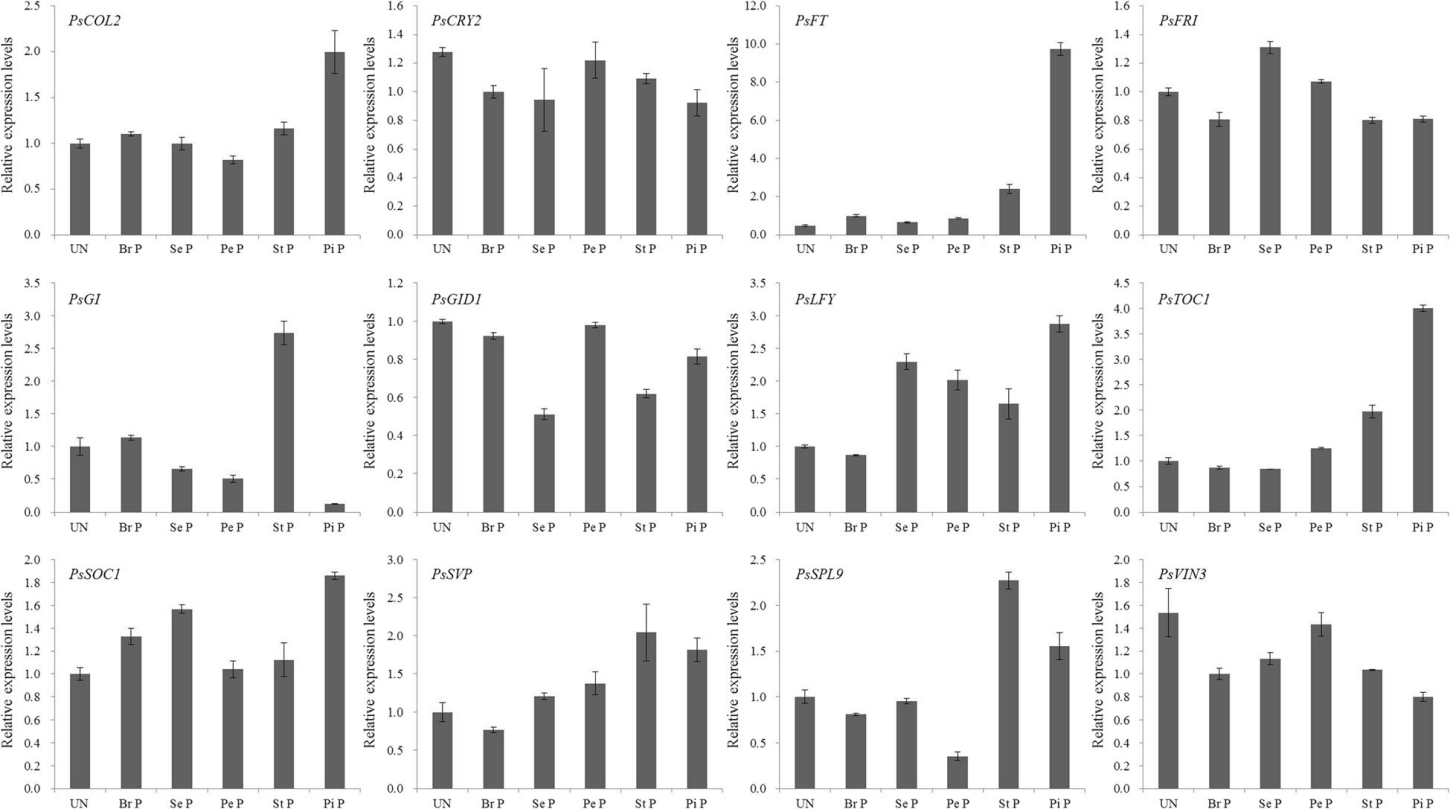
The expression levels of 12 important flowering genes in different primordium buds of ‘Ziluo Lan’. UN, Br P, Se P, Pe P, St P, and Pi P represent buds at the following stages: undifferentiated, bract primordium, sepal primordium, petal primordium, stamen primordium, and pistil primordium, respectively

**Fig. 7:**
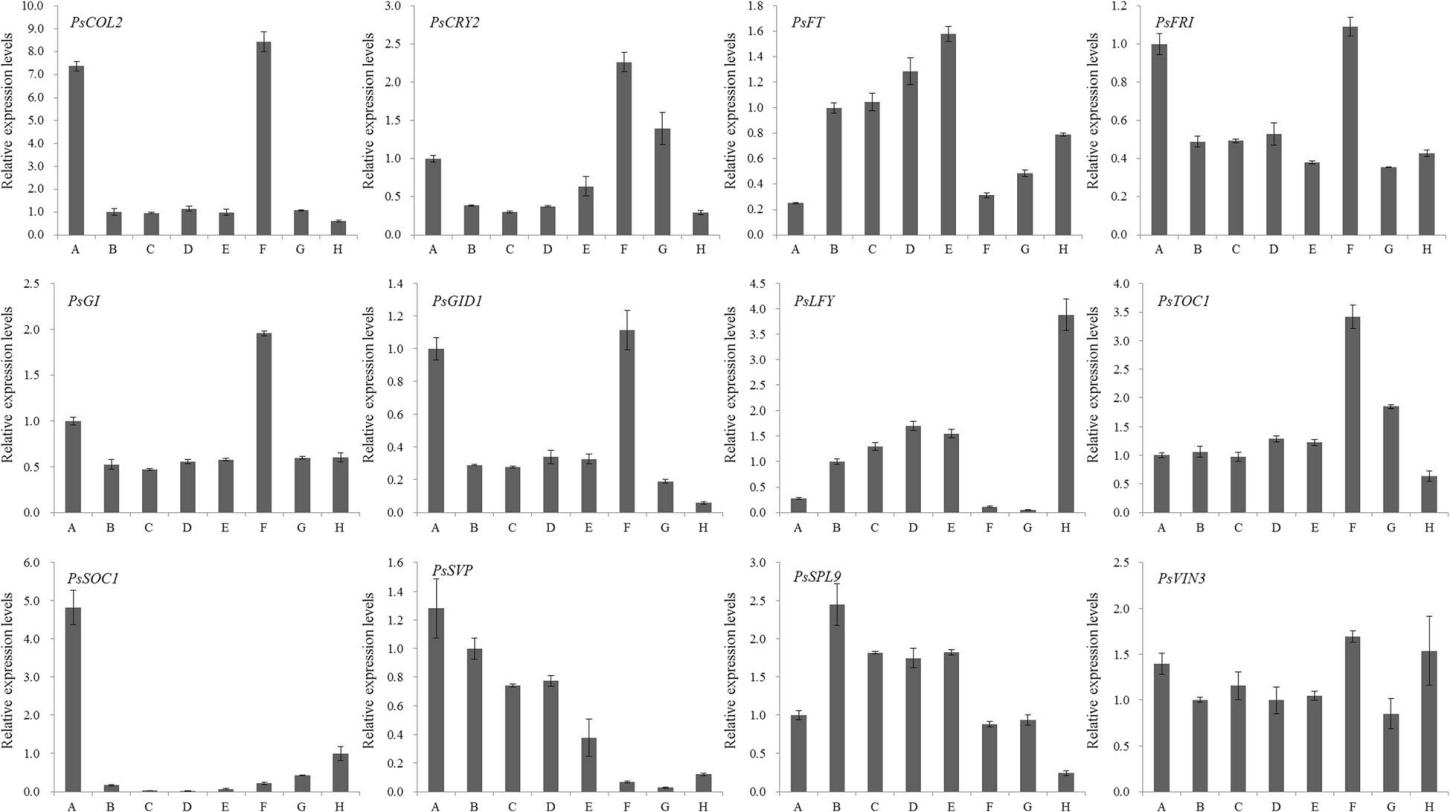
The expression levels of 12 important flowering genes in eight flowering process buds of ‘Ziluo Lan’. A-H represent stages of bud sprouting, leaflet emerging, flower bud emerging phase, flower bud clearly exposed with leaf appearance, small bell-like flower bud, big bell-like flower-bud, bell-like flower-bud extending, and color exposed, respectively

In order to investigate whether the above genes played roles in flowering regulation, the key DEGs and previously reported key flowering time genes from the five pathways were chosen for gene expression analysis in different stages of differentiated primordium and developing buds (Figs. 6 and 7). Except for *FT*, *GI*, and *TOC1*, which were only highly expressed in the buds of stamen or/and pistil primordium stages, long day pathway genes including *COL2*, and *CRY2*, flowering integrator genes *SOC1*, *LFY*, and *SVP*, floral repressor gene *FRI*, vernalization pathway gene *PsVIN3*, gibberellin gene *GID1*, and aging pathway gene *SPL9* were all highly expressed in buds of different stages of differentiated primordium. *PsGI* was highly expressed in the bud at stamen primordium stages (Fig. 6). These results indicated that all 12 genes may regulate bud differentiation, and that the time of regulation was different.

The expression patterns of the above genes were also detected in the buds from stage A (bud swelling) to stage H (color exposing) to detect the function of regulating flowering again. Generally, stages A to E are very important for tree peony flowering, especially for flowering of the forcing culture tree peony. Photoperiod related genes, such as *COL2*, *CRY2*, *GI*, and *TOC1*, and gibberellin gene *PsGID1* had extremely high expression in big bell-like flower buds. Flowering integrator genes *FT* and *LFY* were highly expressed in the buds at key stages (A to E, and H) (Fig. 7). Flowering repressor genes *PsFRI* and *PsSVP* had low expression in buds at stages G and H and had moderate expression in the buds from stages A to F (big-bell like stage) (Fig. 7); these genes are suspected to repress tree peony flowering. *PsSPL9* had higher expression in the bud from stages A to G and may also take part in flowering regulation and bud development in tree peony (Fig. 7). *PsVIN3* also showed high expression in the eight different developmental buds (Fig. 7). The expression of *SOC1* was highest in the sprouting bud and then decreased sharply and was slightly up-regulated in the bud from stages F to H (Fig. 7). These results suggested that *PsSOC1* regulated flowering before bud swelling. Above all, long day, GA, age, and vernalization pathways were shown to be important for the flowering induction pathway in tree peony. The *COL2*, *CRY2*, *GI*, *TOC1*, *PsGID1*, *FT*, *LFY*, *PsFRI*, *PsSVP*, *PsSPL9*, *PsVIN3*, and *PsSOC1* genes were the important genes in the flowering induction pathways.

### Expression analysis of key flowering genes in different treated buds

**Fig. 8:**
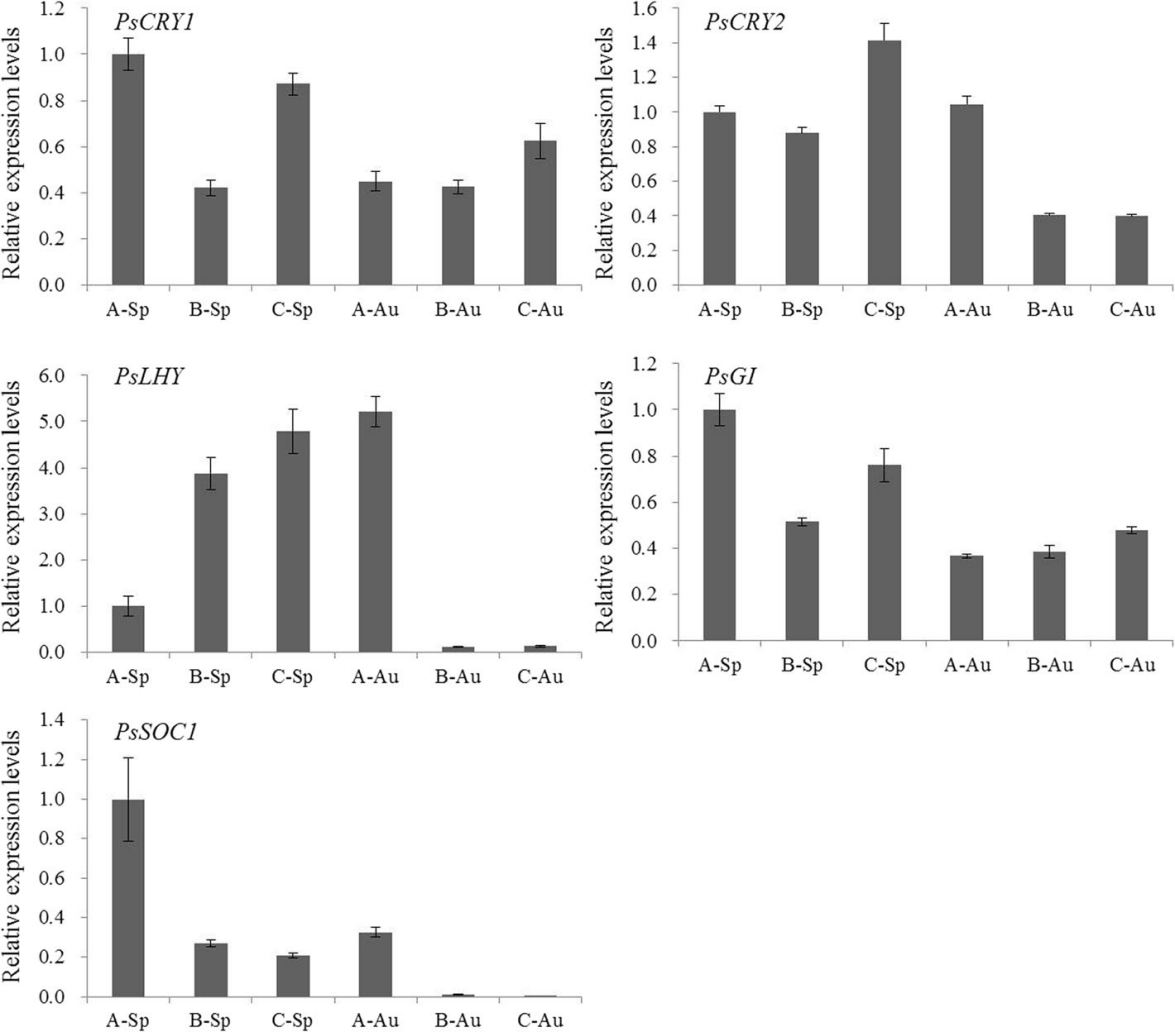
The expression levels of flowering genes *PsCRY1*, *PsCRY2*, *PsLHY*, *PsGI*, and *PsSOC1* in buds at the first three developmental stage of ‘Ziluo Lan’. A, B, and C represent stages of bud sprouting, leaflet emerging, flower bud emerging phase, respectively, and Sp and Au represent spring and autumn, respectively

In order to verify the four flowering induction pathways, treatments were designed for expression analysis of key flowering genes in the four pathways. Tree peony is long day plants, and the differentially expressed unigenes (*Phy A*, *Phy B*, *FKF1*, *CRY*, *GI*, *LHY*, *FT*, *TOC1*, etc.) were mainly involved in the circadian rhythm pathway (Additional file 3: Figure S2). This result indicated that the long day pathway is very important for regulating tree peony autumn flowering or re-blooming. Thus, phytochrome genes *CRY1* and *CRY2*, clock entrainment genes *LHY* and *GI*, and flowering integrator gene *SOC1* were chosen to do expression analysis in the first three developmental stages of buds in spring and autumn. Most of the genes had high expression in the spring buds (Fig. 8). In particular, the expression levels of *PsCRY1* and *PsCRY2* and floral integrator *PsSOC1* were higher in buds in the spring than in autumn. These genes are important for plant flowering [9]. Considering the better flowering quality of spring compared to autumn, this result further indicated that the long day pathway was important for the flowering induction pathway.

**Fig. 9:**
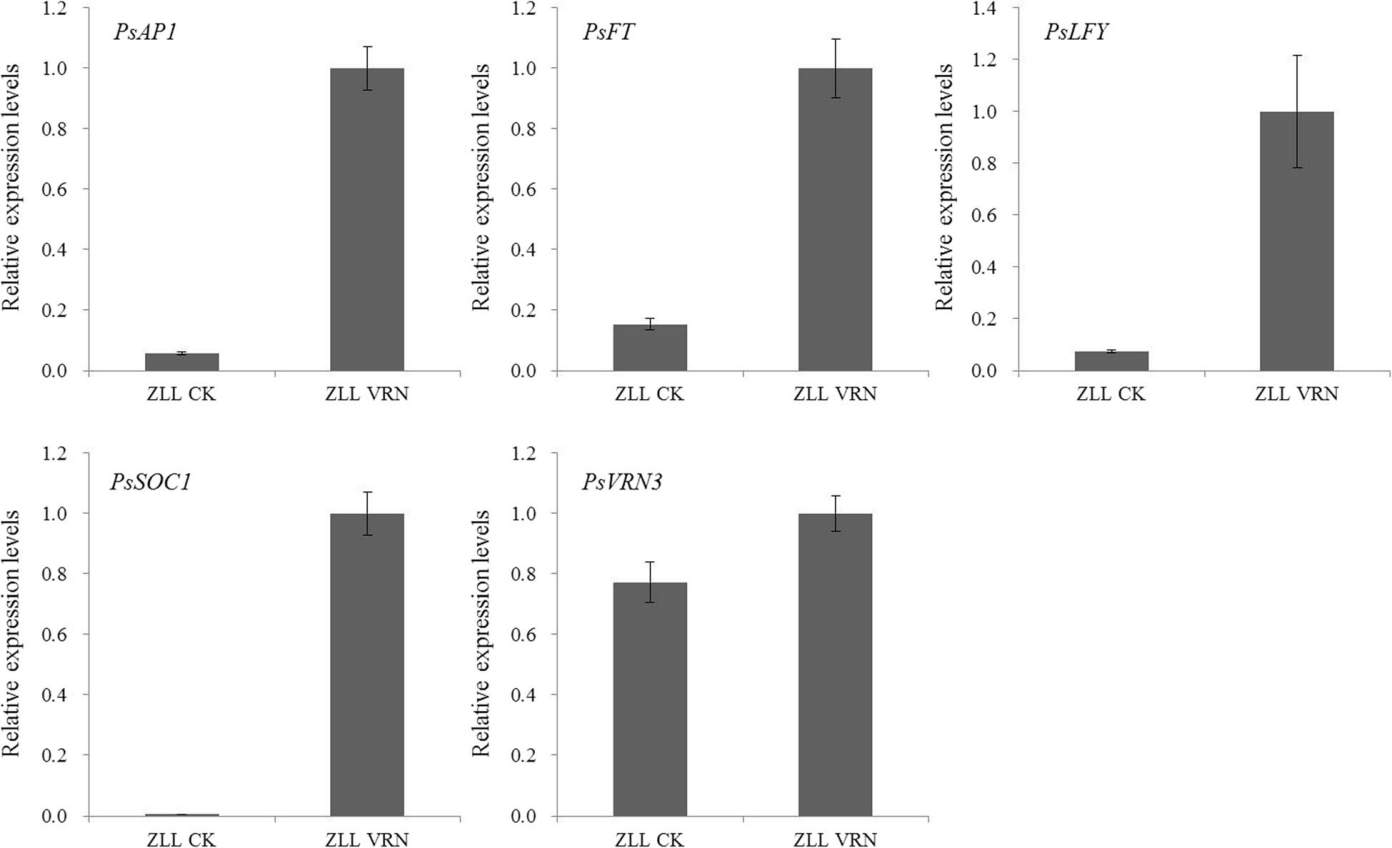
The expression levels of flowering genes *PsAP1*, *PsFT2*, *PsLFY*, *PsSOC1*, and *PsVRN3* in buds with different treatments. CK represents ‘Ziluo Lan’ with no treatment, while VRN represents ‘Ziluo Lan’ with vernalization treatment

The expression levels of *PsAP1*, *PsFT*, *PsLFY*, *PsSOC1*, and *PsVRN3* could be up-regulated by vernalization treatment (Fig. 9). According to the gene function in the model plants, those genes were key genes regulating plant flowering, and higher expression of these genes may be induced by tree peony flowering. These up-regulated genes also play important roles in regulating ‘Ziluo Lan’ re-blooming in autumn.

**Fig. 10:**
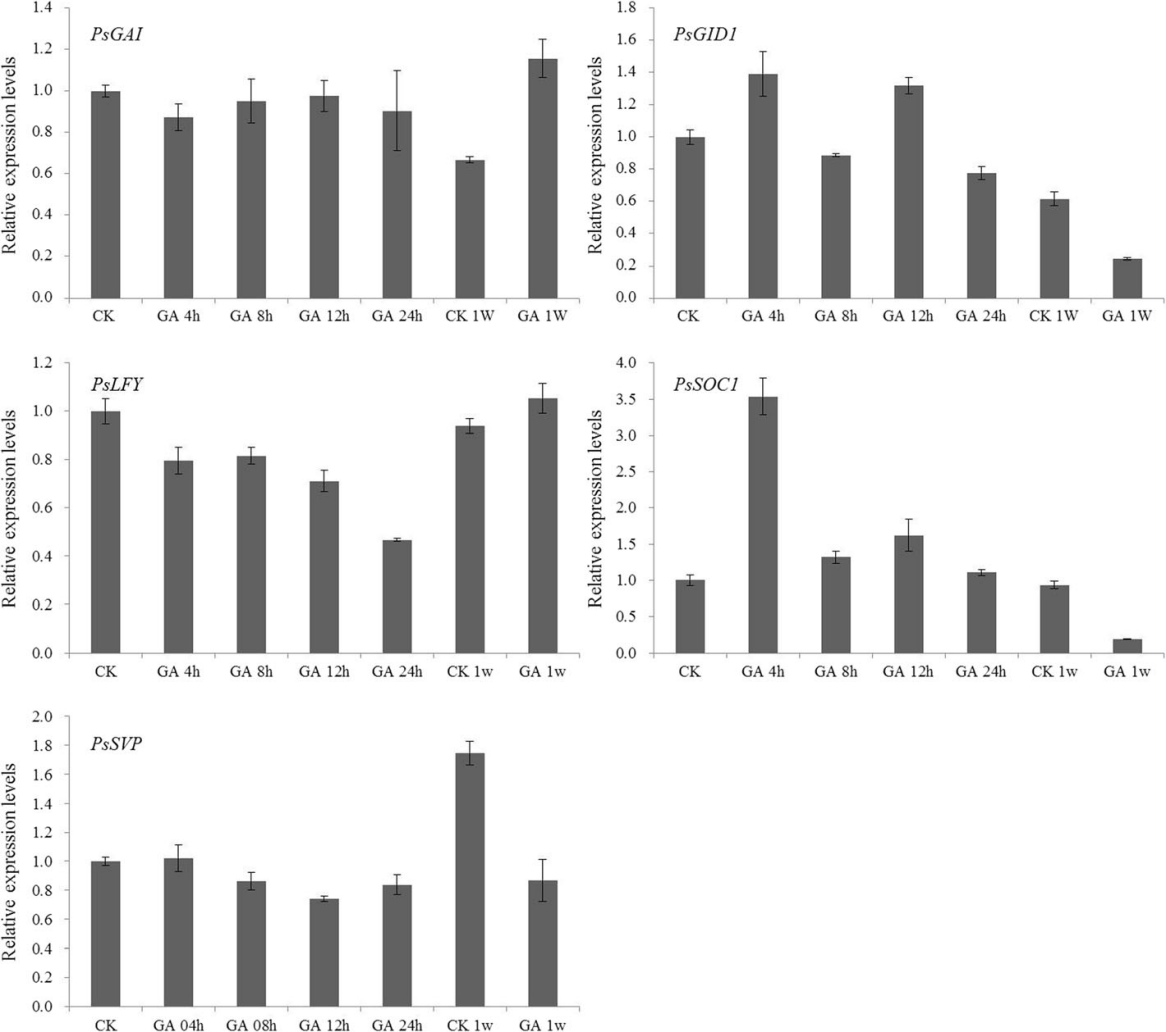
The expression levels of GA synthesis genes *PsGAI* and *PsGID1*, and flowering time genes *PsLFY*, *PsSOC1*, and *PsSVP* in GA_3_ treated buds of ‘Ziluo Lan’. CK and CK 1 W represent buds after 0 h and 1 week without GA_3_ treatment, respectively, while GA 4 h, GA 8 h, GA 12 h, GA 24 h, and GA 1 W represents buds after 4 h, 8 h, 12 h, 24 h with GA_3_ treatment, respectively

GA_3_ treatment results showed that GA_3_ promoted *PsGAI* and *PsLFY* expression in the treated buds after 1 week, and repressed *SVP* gene expression (Fig. 10). The expression levels of *PsGID1* and *PsSOC1* were promoted in buds 4 h after GA_3_ treatment and repressed after 1e week treatment (Fig. 10). *PsGAI* and *PsGID1* are two important GA signaling genes. *PsGID1* is upstream of *PsGAI*, and more *PsGID1* expression will repress *PsGAI* expression [13]. The expression results of the two GA signaling genes were similar with the previous study [13]. Endogenous GA_3_ could promote exogenous GA biosynthesis with 4 h treatment, and more biosynthetic GA induced *PsGID1* expression and repressed *PsGAI* expression (Fig. 10). The expression levels of the flowering time genes were consistent with their functions. Expression results of those genes further validated that tree peony flowering could be induced by the GA pathway in the short day pathway rather than the long day pathway.

### Confirmation of differential expression of floral homeotic genes

**Fig. 11:**
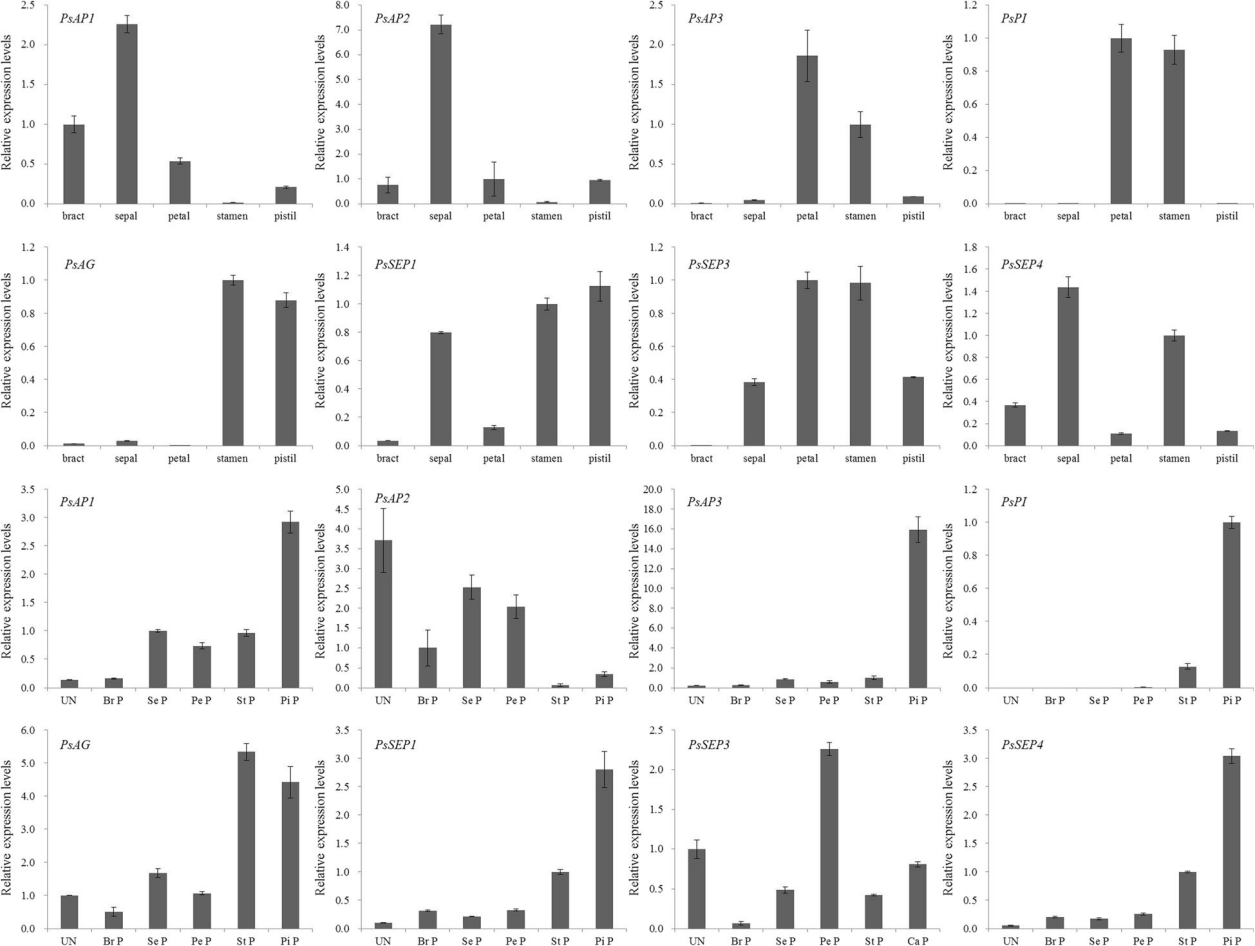
The expression levels of eight floral homeotic genes in five flower parts, and different differentiated primordium stages buds. UN, Br P, Se P, Pe P, St P, and Pi P represent buds at the following stages: undifferentiated, bract primordium, sepal primordium, petal primordium, stamen primordium, and pistil primordium, respectively

The eight identified floral homeotic genes including *AP1*, *AP2*, *AP3*, *PI*, *AG*, *SEP1*, *SEP3*, and *SEP4* were used to confirm their expression in different floral organs and different developmental stages of buds to determine their potential roles in floral organ development. The eight floral homeotic genes displayed distinctive spatial expression patterns in various floral organs (Fig. 11). *AP1* and *AP2* were predominantly expressed in the bract and sepal but had weak expression in petal and pistil and were hardly detected in stamen. In contrast, *AP3* and *PI* had strong expression in petal and stamen, but lower expression in pistil and sepal and were not expressed in bract. *AG* had high expression in stamen and pistil and lower expression in sepal and bract. Although the expression profiles of *SEP1*, *SEP3*, and *SEP4* genes were different, they were expressed in the four whorls of flower organs. The *SEP1* gene was preferentially expressed in sepal, stamen, and pistil; *SEP3* was expressed in the buds of four different flower parts; and *SEP4* had high expression in sepal and stamen. The above results suggested that *AP1* and *AP2* played roles in bract and sepal development; *AP3* and *PI* regulated petal and stamen development; and *AG* took part in stamen and pistil development. In addition, *SEP1*, *SEP3*, and *SEP4* genes regulated development of the four whorls of floral organs.

In the different stages of differentiated flower buds, the expression patterns of *AP1* and *AP2* were opposite, with high expression of *AP1* in buds at the pistil primordium stage and high expression of *AP2* in undifferentiated buds and buds at sepal and petal primordium stages (Fig. 11). Both *AP3* and *PI* had high expression in the bud at the pistil primordium stage (Fig. 11). The *AG* gene was expressed in all undifferentiated and differentiated buds and had especially high expression in the buds at the stamen and pistil primordium stages (Fig. 11). The *SEP1*, *SEP3*, and *SEP4* genes were expressed in all the buds at four different differentiated stages (Fig. 11). These temporal expression results further confirmed that bract and sepal development were due to expression of class A genes such as *AP1* and *AP2*, stamen development was due to the expression of class B genes *AP3*, and *PI*, and class C gene *AG*, and pistil development was due to the expression of class C gene *AG*. Petal development was very complex most likely due to extremely abundant flower types of tree peony. The class E genes *SEP1*, *SEP3*, and *SEP4* regulated the four whorls of floral organs development by interacting with class A, B, or C genes.

## Discussion

Tree peony has large and colorful flowers that are valued globally. However, the short and relatively uniform flowering period is an important hindrance for tree peony production. Forcing cultures are often used to achieve year-round opening of ornamental peonies. However, little genomic information is available for this species, which limits the improvement of forcing culture technology. In this paper, we employed RNA-seq technology on buds of different tree peony cultivars to identify putative genes involved in flowering, floral organ development, and re-blooming. Some important flowering genes were validated by qRT-PCR in buds at different developmental stages or treated buds to determine their function. The flowering induction pathway and the mechanism of flower organ development were proposed. These results will provide a theoretical basis for flowering regulation.

### Flowering habit and period of different tree peony cultivars

Tree peonies have a long cultivation history in China and have been introduced to many countries starting in the Tang Dynasty [23]. Now, more than 2000 tree peony cultivars have been cultivated by long-term artificial selection and cross breeding [1, 2, 23]. Almost all of the cultivars flower once in the spring. Meanwhile, most spring flowering cultivars are mid-season peonies, and a smaller number of cultivars are early- and late-flowering [23]. The flowering habit of peonies has been artificially limited to extend the flowering period. However, some cultivars flower more than twice a year. American-group hybrid (*P. lutea* x *P. suffruticosa*) ‘High Noon’ not only flowers in spring, but also tends to re-bloom in autumn, and sometimes flowers again after the first flowering and before re-blooming in autumn [16]. A few intersectional hybrids (*P. suffruticosa* x *P. latiflora*) also have re-blooming character, with a late and long flowering period. The wild species *P. delavayi*, *P. lutea*, and *P. potaninii* are late-flowering, normally flowering in spring and can re-bloom at random in autumn [12]. Some Chinese cultivars ‘Ziluo Lan’, ‘Bingzhao Lanyu’, ‘Chaoyang Hong’ and ‘Aoshuang’ can re-bloom in the autumn [11, 25]. Japanese cultivar ‘Huchuan Han’ and Chinese cultivar ‘Luoyang Hong’ were often used for forcing culture in winter [1, 2]. The reasons for flowering habit and period in peony are unclear. Thus, understanding the mechanisms of regulating flower habit and period of peonies benefits forcing culture and breeding for flowering timing.

The large genome of tree peony (about 12.5 Gb) coupled with a large amount of repetitive DNA has prevented genome sequencing projects in this species. De novo RNA-seq is often used to identify functional genes [22, 26]. Thus, four tree peony cultivars with different flowering habit and period and one wild species were used to do RNA-seq to decipher the mechanism of flowering regulation. Four hundred fifty four GS-FLX has many advantages for assembling and characterizing the gene space of a non-model species [27]. In this work, 31,505 contigs were assembled into 29,275 unigenes and 22,823 unigenes were annotated by NCBI-NR database. Compared with similar work reported in tree peony, the numbers and annotation information of unigenes was larger [15]. The average length of unigenes was longer than those reported in *P. suffruticosa*, *Larix leptolepis*, and *Epinephelus coioides* [15, 16, 27]. The highest matched species of the annotated sequences was *V. vinifera*, similar to that of *P. ostii* [15]. The sequence quality of 454 GS-FLX was high in our work. Differentially expressed genes (DEGs) were found in different flower habit and period of tree peonies. The largest number of DEGs was found between American cultivar ‘High Noon’ and wild species *P. delavayi*. Different developmental buds of the same cultivar also had a large number of DEGs. These results show that the mechanism of ‘High Noon’ and *P. delavayi* may be different and that flowering is regulated by many important flowering genes.

### DEGs and putative schematic network of flowering induction pathways

The flowering of tree peonies is a response to cues related to light, temperature, and other external influences [1, 2, 12, 13, 25]. Although the analysis of DEGs in re-blooming and non-re-blooming cultivars, or early and late flowering period cultivars has identified some important flowering time genes and re-blooming genes using Illumina HiSeq™ 2000 and Illumina HiSeq™ 2500 platforms [15, 16, 23], the flowering induction pathway in tree peony remains unknown. Eight putative candidate genes of DEGs associated with floral induction, including *PsCO*, *PsGI*, *PsFRI*, *PsVIN3*, *PsGA20ox*, *PsGID1*, *PsSOC1*, and *PsFT*, were found in tree peonies [16]. These genes are involved in photoperiod, vernalization, and GA pathway. In this study, to comprehensively identify the candidate genes putatively implicated in flowering regulation in tree peony, a local BLASTx similarity search was performed against *Arabidopsis* and rice flowering genes from the NCBI database. Some flowering genes, such as *FRI*, *CRY1*, *PHYA*, *TFL*, and *FVE* were identified for the first time in tree peony. At the same time, some important flowering genes including *AG*, *CAL*, *FY*, *LFY*, *HOS*, and *VIN3* were cloned by RACE or RT-PCR in our lab. Floral repressors including *PsMAF1–4*, *PsTFL2*, *PsTOE1–2*, and *PsPIE1* and floral promoters *PsAG* and *PsMAF5* were characterized in our study. Finally, 67 flowering time genes involved in the flowering induction pathway, floral integrators, repressors, promoters, and organ development were obtained (Table 2), representing the most comprehensive report of flowering genes in tree peony.

**Fig. 12:**
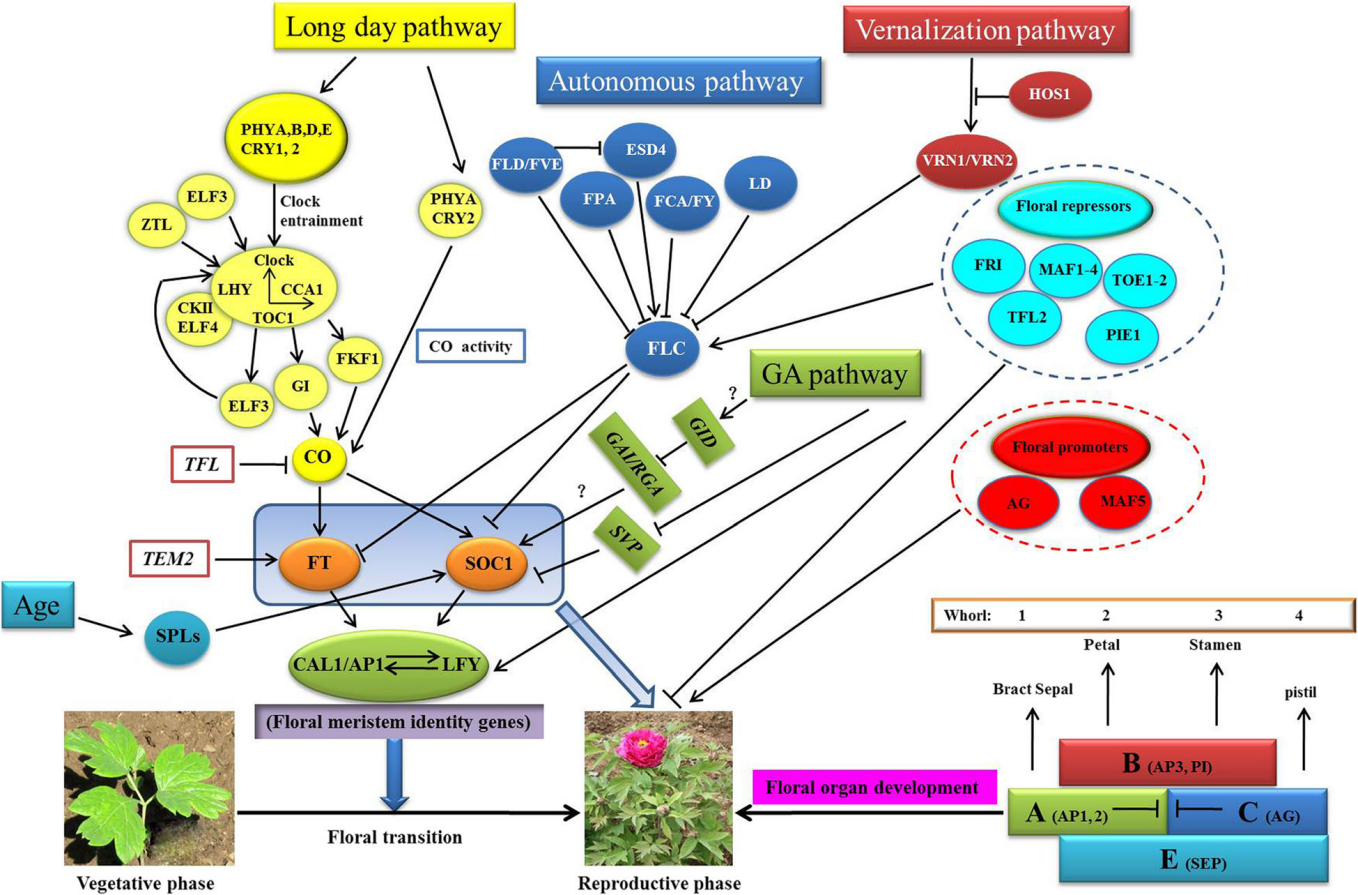
The putative schematic network of flowering induction pathways and floral organ development in tree peony. *Arrows* indicate positive regulation and *bars* indicate negative regulation

In order to construct the schematic network of flowering regulation, the expression patterns of the flowering genes were determined. Expression levels of the flowering-related genes were compared in the buds of four tree peony cultivars, one wild species, and two developmental buds of ‘Ziluo Lan’ to determine the putative schematic network of flowering in tree peony (Additional file 5: Table S2). According to different expression levels of those genes (Additional file 5: Table S2), and their functions in model plants [9], the completed schematic network of flowering induction pathways of tree peony was proposed. In tree peony, five pathways viz. long day, autonomous, vernalization, age, and gibberellin pathway regulated flowering (Fig. 12). However, the genes involved in the vernalization pathway did not show significant changes, except for *PsFRI*, based on DEGs analysis. The vernalization experiment showed that vernalization could significantly increase *PsAP1*, *PsFT*, *PsLFY*, *PsSOC1*, and *PsVIN3* expression. By contrast, the number of DEGs was large in long day and autonomous pathways and it was deduced that long day and autonomous pathways were the two main flowering induction pathways. The expression levels of DEGs, such as *GAI* and *GID1*, in the GA pathway showed significant changes (Additional file 5: Table S2). Combining the results of effects of endogenous GA_3_ on flowering quality of ‘Luoyang Hong’ and the re-blooming mechanism of ‘High Noon’, it was deduced that the GA pathway and vernalization pathway were also important pathways in tree peony [13, 16]. *PdSPL9* played important roles in the juvenile-to-adult phase transition, suggesting that the age pathway was also important in tree peony [12]. Above all, five main pathways, autonomous, long day, vernalization, age, and gibberellin regulated flowering in tree peony.

In order to verify the genetic network of the probable pathways, the important genes of the five pathways and floral integrators, repressors, and promoters were selected to do gene expression analysis in the different differentiated primordium and developmental buds (Figs. 6 and 7); the expression patterns of the above genes further confirmed the reliability of the flowering induction pathways of tree peony. Moreover, these results revealed that the known genetic flowering induction pathways and many critical flowering genes shared a high degree of conservation in tree peony, rice, radish, and *Arabidopsis* [4, 21, 28]. More flowering genes should be identified to improve the genetic network of flowering regulation in tree peony in the future.

### DEGs and the specification of flower organ development

Flower type is an important ornamental character, and there are ten flower types in tree peony. Stamen or pistil petalody results in increased whorls of petals and generates different flower types, which are one of the most important traits for cultivar classification [29]. However, the molecular mechanism of regulated floral organ development is still unclear. The family of MADS-box genes is a major group of regulators controlling floral transition, the specification of floral organ development, and regulating flowering time and other aspects of reproductive development [30, 31]. Our results also identified some members of MADS-box genes, such as *AGLs*, *AP1*, *AP3*, *PI*, and *SEPALLATA1* (*SEP1*), which might participate in the specification of flower organ development (Table 2). The A-function homeotic gene *AP2* was also identified in this study and the C-function homeotic genes *SEP3* and *SEP4* were isolated by RACE and RT-PCR cloning in our lab (Table 2). All of the floral homeotic genes in tree peony were characterized in our study, providing valuable gene resources for flower type investigation.

In addition, the expression levels of eight homeotic genes were detected in the different flower parts and different stages of differentiated primordium and developmental buds to determine the gene function and floral organ development model. The bract covering the outside of the sepal was very large and unique in tree peony and the expression levels of the eight genes were investigated in the bract. The results revealed that eight genes had specific expression levels in five flower parts (Fig. 11). Results suggested that A-function genes including *PsAP1* and *PsAP2* determined sepal development and B-function genes including *PsAP3* and *PsPI* determined petal development. *PsAG* was essential for stamen and pistil development, while *PsSEP3* played important roles in development of the four whorls of floral organs. Development of the second whorl needed cooperated regulation by A-function genes, while third whorl development needed cooperated regulation of B-function genes. Based on these data, the genetic network of floral organ development in tree peony was postulated (Fig. 12). The floral organ development model of tree peony is an ABCE model, consistent with previous studies [32]. The bract development may be determined by A-function genes *PsAP1*, *PsAP2*, or A-function genes + *PsSEP4*. The function of the *PsSEP1* and *PsSEP4* genes should be further investigated, as they are probably essential for abundant flower types and bract development. The alternative splicing of *PhAGL6b* was a key gene regulating specific labellum forming in *Phalaenopsis* [14]. Alternative splicing and variation of floral organ genes is also involved in abundant flower type forming in tree peony (data unpublished). Thus, the genetic regulation network of flower type is very complex. This postulated genetic network could provide a theoretical basis for tree peony flower type breeding.

### The important re-blooming genes of tree peony

Re-blooming is very important for extending the flowering period and directly increasing economic benefits of ornamental tree peonies. Re-blooming genes were extensively investigated in the past 5 years [1, 2, 16, 23, 25]. Four genes, *PsCO*, *PsFT*, *PsGA20ox*, and *PsVIN3* probably play important roles in the regulation of the re-blooming process in tree peonies [16]. The expression patterns of GA biosynthesis and metabolism genes showed that *PsGA20ox*, *PsGA2ox*, and *PsGA3ox* were involved in the bioactive GAs synthesis, instead of directly operating in flowering [13]. The *PsSVP* and *PsSOC1* genes are involved in flowering and vegetative growth of forcing culture tree peonies [1, 2]. *PsCRY2* had higher expression in re-blooming ‘Ziluo Lan’ than that in non-re-blooming ‘Luoyang Hong’ and was increasingly expressed in the bud under long day conditions, compared short day conditions [25]. Previous studies and the expression patterns of the key flowering time genes in re-blooming and non-re-blooming tree peony, different stages of differentiated buds, flowering process, and vernalization experiment in this work, suggested that *PsAP1*, *PsCOL1*, *PsCRY1*, *PsCRY2*, *PsFT*, *PsLFY*, *PsLHY*, *PsGI*, *PsSOC1*, and *PsVIN3* were the candidate re-blooming flowering genes. The most important re-blooming genes should be identified in the future.

## Conclusions

This work presents de novo transcriptome sequencing analysis of tree peony flower development using the 454 GS-FLX platform. A total of 29,275 unigenes were assembled with an average length of 677.18 bp, and 23,332 unigenes were annotated by at least one database among NCBI-NR, Swiss-prot, COG, GO, and KEGG. A total of 67 flowering-related genes were identified in tree peony, and the genetic regulation network of the flowering induction pathways and floral organ development were postulated. Moreover, the genes that regulated re-blooming in tree peony were proposed. Our work provides a theoretical basis for tree peony forcing culture and breeding for flowering period and flower type.

## Methods

### Plant materials and sample collection for transcriptome sequencing

**Fig. 13:**
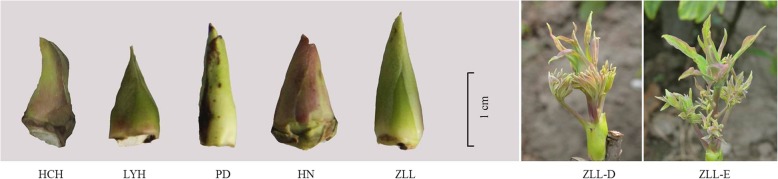
The morphology of the seven samples used for transcriptome sequencing. HCH, LHY, PD, HN, ZLL, ZLL-D, and ZLL-E represent the peony names, ‘Huchuan Han’, ‘Luoyang Hong’, *P. delavayi*’, ‘High Noon’, ‘Ziluo Lan’, clearly exposed buds of ‘Ziluolan’, and small bell-like flower bud of ‘Ziluolan’, respectively

In this work, transcriptome sequencing and gene expression analysis were performed on seven samples of tree peonies, including four cultivars and one wild species (Fig. 13). All of the cultivars and one wild species were introduced from Luoyang Tree Peony Gene Bank, China (there are no Genbank numbers, and only cultivar names in the Tree Peony Gene Bank), and grown in the Institute of Vegetables and Flowers Chinese Academy of Agricultural Sciences. Of these, ‘Huchuan Han’ with mid-flowering type, was from Japanese cultivars; ‘High Noon’ with late-flowering type, was from American cultivars; and ‘Luoyang Hong’, ‘Ziluo Lan’, and *P. delevayi* were Chinese cultivars or wild species and their flowering times were early-, mid- and late-flowering type, respectively. The cultivar names were referred to Li et al. [33] and the wild species was named by Abbe’Delavay for the first time [3, 33]. The buds of the five samples (four cultivars and one wild species) were collected on 23-7-2012, and the other two samples, viz. clearly exposed buds and small bell-like flower bud of ‘Ziluo Lan’ were collected on 22-8-2012 and 12-9-2012, respectively. The seven samples for transcriptome sequencing were immediately frozen in liquid nitrogen and stored at − 80 °C. ‘Huchuan Han’ can re-bloom in winter by forcing culture in Japan. ‘High Noon’ can re-bloom at autumn in natural conditions, while *P. delevayi* can re-bloom in autumn at random, and ‘Ziluo Lan’ can re-bloom in autumn with leaflet removal and GA_3_ application [2]. ‘Luoyang Hong’ was always used for tree peony forcing culture in winter; however, it could not easily re-bloom in autumn [1].

### Plant treatment and material collection

In order to investigate the floral inductive pathways, floral organ development model, and mechanism of re-blooming at autumn, ‘Ziluo Lan’ was selected to do different treatments. Detailed treatments were as follows:

1. Sixty plants were selected to study the GA pathway. Leaves and extra buds were removed from half of the plants (only one to two buds were left for re-blooming) on 23-7-2012, and the remaining plants were as a control where only extra buds were removed. GA_3_ treatment was applied to buds at 6-8-2012, 8-8-2012, and 10-8-2012, respectively, to promote bud development and flowering. Buds were collected on 6-7-2012 and after GA treatment for 4 h and 1 week.
2. Bud development was divided into eight stages, and the morphological developmental buds of ‘Ziluo Lan’ are in Additional file 6: Figure S4. A: Bud sprouting. In this stage flower buds tip emerged but was still covered by the scale. B: Leaflet emerging. The leaflet emerged but remained incurved. C: Flower bud emerging phase. Flower bud emerged and petiole extended, while the leaflet is still incurved. D: Flower bud clearly exposed with leaf appearance. Flower bud grows and its height is higher than that of leaflets. E: Small bell-like flower bud. Flower buds like a small bell. The leaves began unfolding and petiole opened outward. F: Big bell-like flower-bud. Typical characteristics in this stage are that the flower bud enlarges, sepals become flat, and the leaf unfolds completely. G: Bell-like flower-bud extending. Enlarging flower bud turns large and tight. H: Color exposed. The colorful petal is exposed accompanied by loose and soft flower bud. Eight buds at different developmental stages were collected from 10 to 3-2013 to 28-4-2013. Buds at stages A, B, and C were also collected on 2-8-2012, 6-8-2012, and 9-8-2012, respectively, in the autumn by forcing culture treatments, to conduct re-blooming gene expression analysis. All the materials were cultivated in the field of the experimental base of the Institute of Vegetables and Flowers, Chinese Academy of Agricultural Sciences.
3. Another 60 plants were selected to study vernalization. They were potted on 1-9-2012 and after 1 month of growth, half of the plants were stored in the refrigerator at 4 °C for one week; the remaining plants were stored in a 25 °C greenhouse. Buds with vernalization treatment and control (CK) were collected. The morphology of buds could be seen in Additional file 7: Figure S5.

All three treatment buds were replicated from 2013 to 2014 and 2015–2016 for biological replicates.

(4) Five different differentiated primordium stages of buds were collected from 2013 to 6 to 2013–10 (Additional file 8: Figure S6).
(5) Five whorls of floral organs were collected on 2-5-2013.

### RNA extraction, construction of the cDNA library, and transcriptome sequencing

For transcriptome sequencing, total RNA was extracted from seven samples using phenol-chloroform extraction. Concentration and purity of the total RNA was determined using a Nanodrop 1000 (Thermo Scientific, USA). The mRNA was isolated and concentrated according to the instructions for the PolyATtract® mRNA Isolation Systems (Promega, USA), and RNeasy RNA Cleaning Kit (QIAGEN, Germany), respectively. The mRNA integrity and quantity were assessed using an Agilent BioAnalyzer 2100 (Agilent Technologies, Santa Clara, CA). The first-strand cDNA and double-stranded cDNA (dscDNA) synthesis and dscDNA treatments were as in Zhang et al. [27]. Finally, the cDNA samples were processed with Roche 454 Genome Sequencer (GS) FLX Titanium General DNA Library Preparation Kit (Roche), following the manufacturer’s instructions. Sequencing was carried out using a Roche 454 GS-FLX instrument. All the obtained data are available at the NCBI Short Read Archive (https://trace.ncbi.nlm.nih.gov/Traces/sra_sub/sub.cgi, accession number: SRX863944).

### De novo transcriptome assembly and annotation

Raw data generated from 454 sequencing were preprocessed to remove the sequences of adapters, ambiguous nucleotides (‘N’ in the end of reads) and low-quality sequences using LUCY software [34] and Seq-clean programs (http://sourceforge.net/projects/seqclean/). The screened high-quality sequences were subjected to de novo assembly using the Contig Assembly Program, CAP3, under default parameters [35]. Then, CD-HIT-EST was used to remove redundancy and retain the longest possible contigs. The short redundant contigs were removed and the remaining contigs composed the final unigenes for further analysis.

For annotation, the final unigenes were searched against the NCBI non-redundant (NR) protein database (2013.05) using BLASTx, with a cut-off E-value of 10^− 5^ at first. Then, the final unigenes were used for BLASTx searches against the uniProt/Swiss-Prot protein database (2013.05). The unigene sequences were also aligned to the COG database (*e* value < 1.00E-05) to predict and classify functions. To understand the functional classification of the unigenes, gene ontology (GO) analysis was conducted on the annotated sequences using the Blast2GO Program [36]. In addition, to gain an overview of gene pathway networks, we carried out the Kyoto Encyclopedia of Genes and Genomes (KEGG) annotations based on the KEGG database.

### Differentially expressed genes (DEGs) analysis

The Reads per Kilobase per Million mapped reads (RPKM) method was used to calculate the gene expression level [37]. Based on “the significance of digital gene expression profiles”, differentially expressed genes (DEGs) between samples and their corresponding *P*-value were determined using methods described by Audic and Claverie [38]. The threshold of the *P*-value in multiple tests was determined by the value for the false discovery rate (FDR) [39]. FDR ≥ 0.001 and the absolute value of log_2_Ratio ≥ 1 were used as the threshold to judge the significance of the gene expression differences.

### Quantitative real time PCR verification and expression analysis

The extraction of total mRNA from different developmental stage buds, tissues, and organs, and buds with different treatments, mRNA purification, and cDNA synthesis were performed according to previously reported methods [1, 40]. The DEGs and gene function prediction were performed by quantitative real time PCR (qRT-PCR). The 25 gene-specific primers were designed by primer 6.0 and the detailed information is shown in Table 3. The qRT-PCR program was outlined in Wang et al. [1, 2]. Relative expression levels of the candidate genes were calculated by normalizing to the reference gene *ACTIN* [1]. The qRT-PCR reaction was performed in three biological replicates, and three technical repetitions were performed for each replicate.

**Table 3:** List of primers for expression analysis of flowering genes

Primer name	Sequences of the primers
RTAG-2F	5′-CAGGCAAATGTTGGGTGA-3’
RTAG-2R	5′-TGCTGGGCTCTTTCGTTC-3′
RTAP1-1F	5′-AGAAGAAGGAAAGGGCAATC-3’
RTAP1-1R	5′-TTCCTCCTCACTTCTGTTGG-3′
RTAP2-2F	5′-CACGATGAATCCGATGACG-3′
RTAP2-2R	5′-GAAACCTCCACCGACTTGC-3’
RTAP3.2-1F	5′-TGGTGGAGAATGAGGGAG-3′
RTAP3.2-1R	5′-GGCGGAAAGCATACAAAT-3’
RTCOL1-1F	5′-AGGGCATTCAGTGAAGGAG-3’
RTCOL1-1R	5′-CCTACGCTCTTCAGTGGTG-3’
RTCOL2-3F	5′-GAGGCAAGAGTCCTAAGATACAG-3’
RTCOL2-3R	5′-AACCGCCCTTTGATTCGTG-3′
RTCOL4-2F	5′-TTGGTGAACGGAGGTGGT-3’
RTCOL4-2R	5′-TGAACTGCTGGATGATTTGT-3′
RTCOL11-1F	5′-GAAAAGAGGTGGAGACGAAG-3’
RTCOL11-1R	5′-AGACCACGGGACCACTTGA-3’
RTCRY1-1F:	5′-ACAACTTTCTCGGCATTCT-3′
RTCRY1-1R	5′-CAGCCTTTCTACGGTTCTT-3’
RTCRY2-1F	5′-CGTGCGAATAAAGCAGATA-3’
RTCRY2-1R	5′-GAAACAAAGGTATCGGGAG-3’
RTFRI-2F	5′-TCTTGCCACATTCGGTATT-3’
RTFRI-2R	5′-TCAGACAGGTCAAGGGAGC-3′
RTFT-2F	5′-CCAAGCGACCCAAACCTA-3′
RTFT-2R	5′-CGCCAACCTGGAGTGTAA-3′
RTGAI-1F	5′-GAGTATGCTGTCCGAGTTCA-3’
RTGAI-1R	5′-CAGGAGCAAGGAACGAAT-3’
RTGI-1F	5′-TAACCGCCCAATCTACAAG-3’
RTGI-1R	5′-ATTTTCCCACAACACCGCTG-3’
RTGID1-1F	5′-TGAAGAACCTCCACCAAG-3’
RTGID1-1R	5′-CCACAAGACGACGACAAA-3′
RTLFY-1F	5′-ATGAGAAGGAAGGAGGGGATG-3’
RTLFY-1R	5′-CTTTGGCAATGGTCTGAACT-3’
RTLHY-2F	5′-GCAGTAACAGCGAGTGAGGT-3′
RTLHY-2R	5′-TTGCGGTAATACTTGTCGTGAG-3′
RTSEP1-1F	5′-TGAGCGTCAACTGGAAACAT-3
RTSEP1-1R	5′-AGCAAGCTGATCGAGCATAT-3’
RTSEP3-1F	5′-TTGCGATGCGGAGGTTG-3’
RTSEP3-1R	5′-CCAAGGTCCTCACCAAGAAG-3’
RTSEP4-1F	5′-CTCTAACCGTGGGAAACTC-3’
RTSEP4-1R	5′-ACCTCTACCCTTGCCTTG-3′
PsqSOC1-1F	5′-CCAATGTCCGAGCAAGAAAG-3’
PsqSOC1-1R	5′-CCGTGCTTCTCGCATAACAT-3’
RTSPL9-1F:	5′-GGTTTTGCCAGCAGTGTAGC-3’
RTSPL9-1R	5′-AGTCCATCAGAAAGCCTCCA-3’
RTSVP1-1F	5′-CGATGTTGAGCAAGGAGGTT-3’
RTSVP1-1R	5′-GCTCTAAATCAGCAGCGACA-3’
RT-TOC1-1F	5′-AACTTGCGGCGTATTCCT-3′
RT-TOC1-1R	5′-ATGCGTCTCCTTCTCCAC-3’
RTVIN3-2F	5′-GCAATCCAACGGAAGAAAGT-3’
RTVIN3-2R	5′-AAGCAGCACAGCAGTAACCTC-3′

## Additional files

Additional file 1: **Figure S1.** Sequence length distribution of the unigenes assembled from bud transcriptome sequencing. The horizontal and vertical axes show the size and the number of the unigenes, respectively. (JPG 29 kb)

Additional file 2: **Table S1.** The differentially expressed genes (DEGs) involved in the different pathways. (XLSX 77 kb)

Additional file 3: **Figure S2.** The unigenes involved in the plant circadian rhythm in bud of tree peony. The genes in red were found by our transcriptome sequencing. (PNG 19 kb)

Additional file 4: **Figure S3.** The differentially expressed genes based on comparisons of any two samples in bud transcriptome sequencing in tree peony. (JPG 186 kb)

Additional file 5: **Table S2.** The expression level analysis of the flowering-related genes in the seven samples by the Reads per Kilobase per Million mapped reads (RPKM) method. (XLSX 35 kb)

Additional file 6: **Figure S4.** The morphological characters of buds at eight different developmental stages of ‘Ziluo Lan’. A: Bud sprouting. In this stage flower buds tip emerged but was still covered by the scale. B: Leaflet emerging. The leaflet emerged and remained incurved. C: Flower bud emerging phase. Flower bud emerged and petiole extended, while the leaflet is still incurved. D: Flower bud clearly exposed with leaf appearance. Flower bud grows and its height is higher than that of leaflets. E: Small bell-like flower bud. Flower bud like a small bell. The leaves began unfolding and petiole opened outward. F: Big bell-like flower-bud. Typical characteristics in this stage are that flower bud enlarges, sepals become flat, and leaf unfolds completely. G: Bell-like flower-bud extending. Enlarging flower bud turned large and tight. H: Color exposed. The colorful petal is exposed accompanied by a loose and soft flower bud. (JPG 70 kb)

Additional file 7: **Figure S5.** The morphological characters of bud with or without vernalization. CK represents ‘Ziluo Lan’ with no treatment, while VRN represents ‘Ziluo Lan’ with vernalization treatment. (JPG 12 kb)

Additional file 8: **Figure S6.** The morphological characters of buds at six different differentiated primordium stages. UN, Br P, Se P, Pe P, St P, and Pi P represent buds at the following stages: undifferentiated, bract primordium, sepal primordium, petal primordium, stamen primordium, and pistil primordium, respectively. (JPG 126 kb)

## Data Availability

All data generated or analyzed during this study are included in this published article and its supplementary information files. The nucleotide sequences of raw data from this study were submitted to the NCBI sequence read Archive (SRA) under the accession number SRX863944.

## Abbreviations

*AGL*: *AGAMOUS-LIKE*
*AP1*: *APETALA1*
*CKII*: *Casein Kinase II*
*CO*: *CONSTANS*
COG: Clusters of Orthologous Groups
DEGs: Differentially expressed genes
*FLC*: *FLOWERING LOCUS C*
*FRI*: *FRIGIDA*
*FT*: *FLOWERING LOCUS T*
*GA*: Gibberellins
GO: Gene ontology
*HCH*: Huchuan Han
*HN*: High Noon
KEGG: Kyoto Encyclopedia of Genes and Genomes
*LFY*: *LEAFY*
*LYH*: Luoyang Hong
NR: NCBI non-redundant protein
*PD*: *Paeonia delavayi*
*PHYE*: *Phytochrome E*
*SEP1*: *SEPALLATA1*
*SOC1*: *SUPPERSSOR OF CONSTANS OF OVEREXPRESSION1*
*SPL*: *Squamosa promoter binding protein like*
*SPY*: *SPINDLY*
*SVP*: *SHORT VEGETATIVE PHASE*
*VIN3*: *VERNALIZATION INSENSITIVE 3*
*ZLL D*: Bud at stage D of ZLL
*ZLL E*: Bud at stage D of ZLL
*ZLL*: Ziluo Lan
