# Supplementary figures and images for: De novo sequencing of tree peony (Paeonia suffruticosa) transcriptome to identify critical genes involved in flowering and floral organ development
Source: BMC Genomics. 2019 Jul 11;20:572. doi: 10.1186/s12864-019-5857-0 (PMC6624964; doi:10.1186/s12864-019-5857-0)

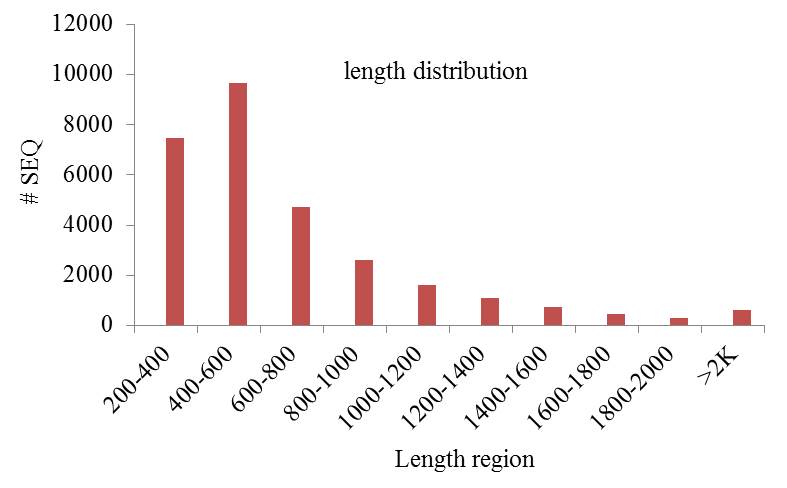

Supplement: Supplementary file 1 — Figure S1. Sequence length distribution of the unigenes assembled from bud transcriptome sequencing. The horizontal and vertical axes show the size and the number of the unigenes, respectively. (JPG 29 kb) [file 12864_2019_5857_MOESM1_ESM.jpg]

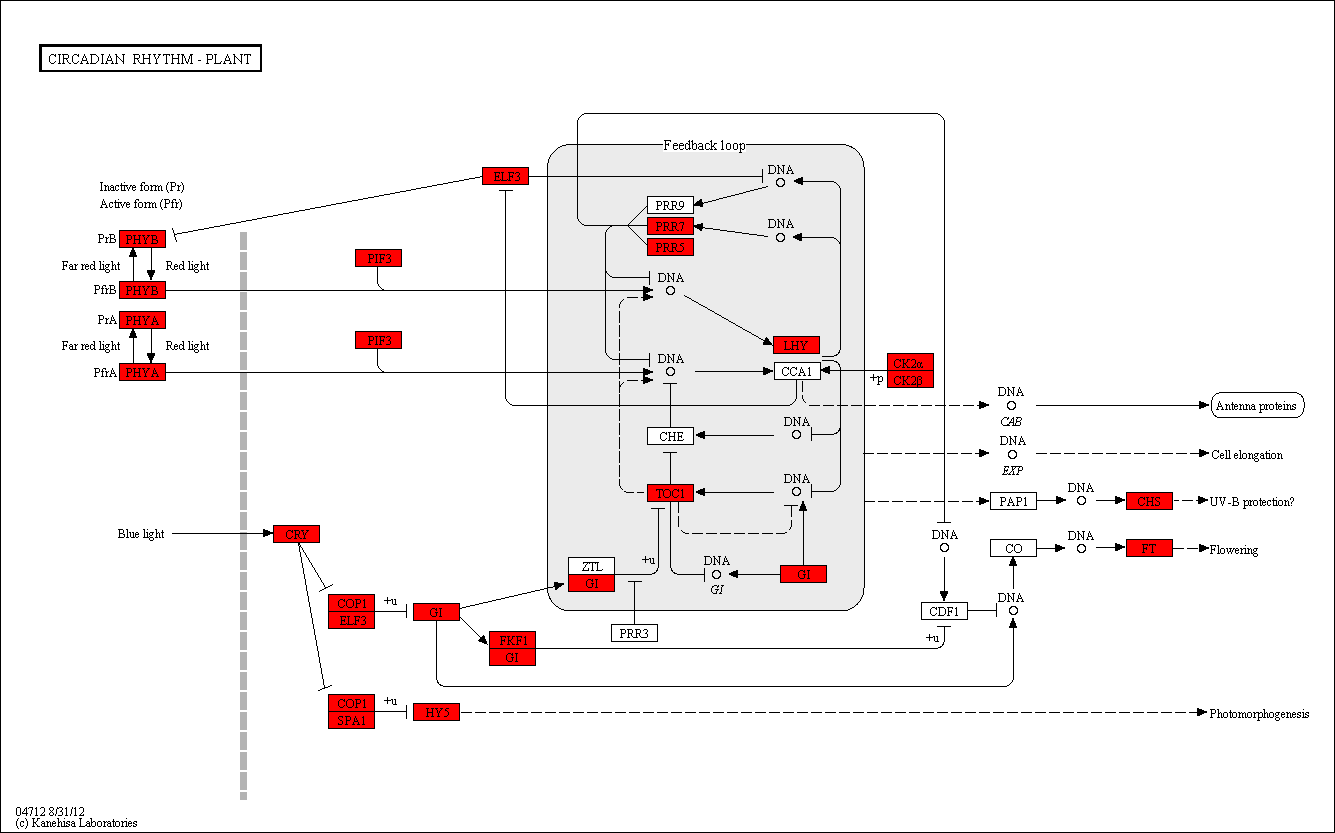

Supplement: Supplementary file 3 — Figure S2. The unigenes involved in the plant circadian rhythm in bud of tree peony. The genes in red were found by our transcriptome sequencing. (PNG 19 kb) [file 12864_2019_5857_MOESM3_ESM.png]

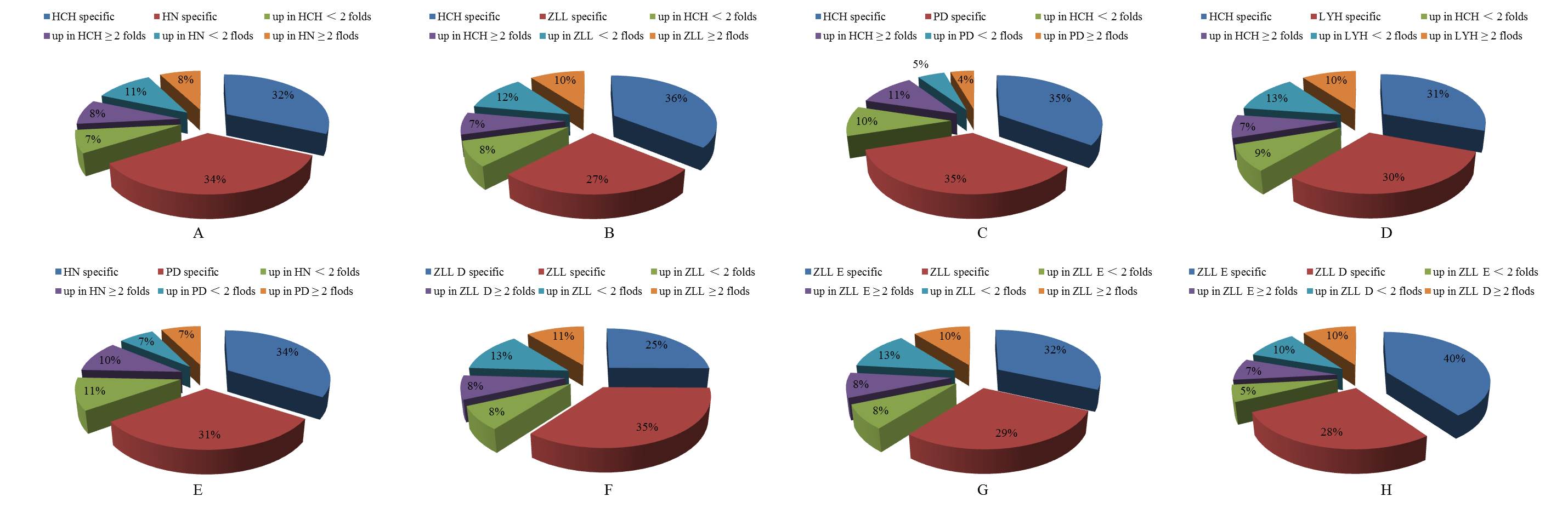

Supplement: Supplementary file 4 — Figure S3. The differentially expressed genes based on comparisons of any two samples in bud transcriptome sequencing in tree peony. (JPG 186 kb) [file 12864_2019_5857_MOESM4_ESM.jpg]

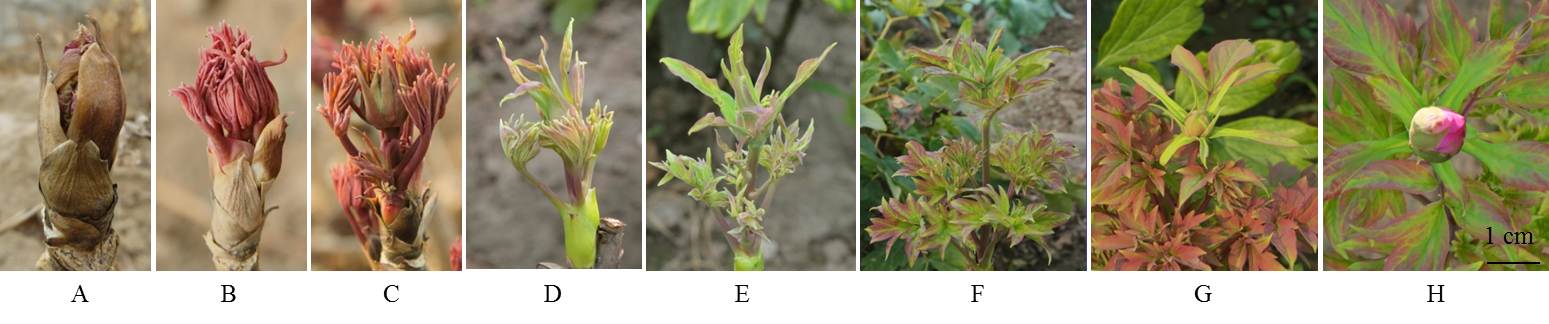

Supplement: Supplementary file 6 — Figure S4. The morphological characters of buds at eight different developmental stages of ‘Ziluo Lan’. A: Bud sprouting. In this stage flower buds tip emerged but was still covered by the scale. B: Leaflet emerging. The leaflet emerged and remained incurved. C: Flower bud emerging phase. Flower bud emerged and petiole extended, while the leaflet is still incurved. D: Flower bud clearly exposed with leaf appearance. Flower bud grows and its height is higher than that of leaflets. E: Small bell-like flower bud. Flower bud like a small bell. The leaves began unfolding and petiole opened outward. F: Big bell-like flower-bud. Typical characteristics in this stage are that flower bud enlarges, sepals become flat, and leaf unfolds completely. G: Bell-like flower-bud extending. Enlarging flower bud turned large and tight. H: Color exposed. The colorful petal is exposed accompanied by a loose and soft flower bud. (JPG 70 kb) [file 12864_2019_5857_MOESM6_ESM.jpg]

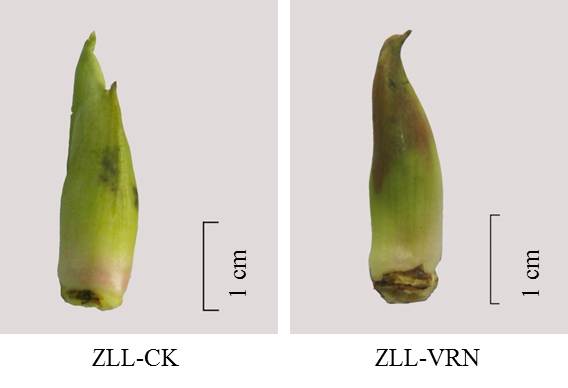

Supplement: Supplementary file 7 — Figure S5. The morphological characters of bud with or without vernalization. CK represents ‘Ziluo Lan’ with no treatment, while VRN represents ‘Ziluo Lan’ with vernalization treatment. (JPG 12 kb) [file 12864_2019_5857_MOESM7_ESM.jpg]

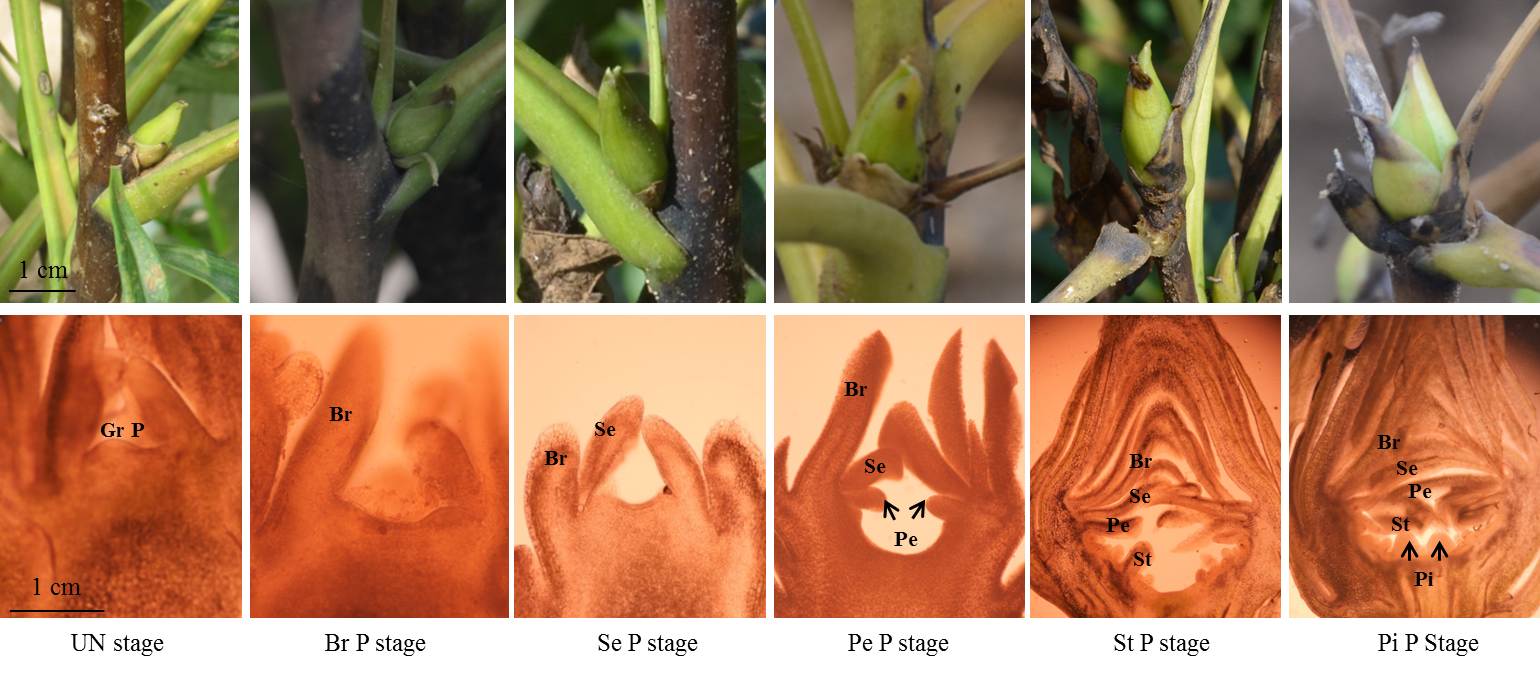

Supplement: Supplementary file 8 — Figure S6. The morphological characters of buds at six different differentiated primordium stages. UN, Br P, Se P, Pe P, St P, and Pi P represent buds at the following stages: undifferentiated, bract primordium, sepal primordium, petal primordium, stamen primordium, and pistil primordium, respectively. (JPG 126 kb) [file 12864_2019_5857_MOESM8_ESM.jpg]
